# The *Saccharomyces* killer toxin K62 is a protein of the aerolysin family

**DOI:** 10.1128/mbio.01425-25

**Published:** 2025-11-11

**Authors:** Jack W. Creagh, Michael Rolfsmeier, Kasen J. Evans, Rodolfo Bizarria, David C. Reetz, Tanner J. Badigian, Lance R. Fredericks, Ava M. Hasenoehrl, Abigail P. Brown, Brayden M. Graves, Connor K. Alexander, Andre Rodrigues, Eric P. Stoffregen, Jagdish Suresh Patel, F. Marty Ytreberg, Paul A. Rowley

**Affiliations:** 1 Department of Biological Sciences, University of Idaho 123394, Moscow, Idaho, USA; 2 Department of Pharmaceutical Sciences, School of Pharmaceutical Sciences of Ribeirão Preto, University of São Paulo, Ribeirão Preto, Brazil; 3 Physical, Life, Movement & Sport Sciences Division, Lewis-Clark State College 4522, Lewiston, Idaho, USA; 4 Department of Chemical and Biological Engineering, University of Idaho 681878, Moscow, Idaho, USA; 5 Institute for Modeling Collaboration and Innovation, University of Idaho 5640, Moscow, Idaho, USA; 6 Department of Physics, University of Idaho 5640, Moscow, Idaho, USA; 7 Institute of Ocean and Earth Sciences, Universiti Malaya, C308, Institute of Advanced Studies Building, Kuala Lumpur, Malaysia; University of Wisconsin-Madison, Madison, Wisconsin, USA

**Keywords:** yeast, killer toxin, *Saccharomyces*, aerolysin

## Abstract

K62 is an antifungal killer toxin produced by *Saccharomyces paradoxus*, encoded by a double-stranded RNA satellite. The toxin exhibits a unique antifungal activity but lacks sequence homology to other killer toxins, and its antifungal mechanism of action remains unknown. To understand the function of K62, its tertiary structure was predicted using AlphaFold, followed by molecular dynamics simulations to create high-confidence molecular models. These analyses revealed that K62 monomers closely resemble the five-beta-strand domain found in pore-forming aerolysin toxins. Models of K62 oligomers yielded a circular complex and beta-barrel with structural and biochemical similarities to aerolysin-family pre-pores and pores. Consistent with the formation of aerolysin-like pores, recombinant K62 assembled into membrane-associated high molecular weight oligomers (>250 kDa) that were heat- and detergent-resistant. K62 has more than 1,000 uncharacterized sequence homologs, which were mostly found in fungi of the Ascomycota, as well as in the Chytridiomycota, Basidiomycota, plants, and bacteria, with evidence of extensive horizontal gene transfer. Homologs were also identified in pathogenic fungal species, including human and plant pathogens from the *Candida* and *Fusarium* genera, but unlike aerolysins, K62 appeared to be non-toxic to higher eukaryotes. K62 is the first aerolysin family protein discovered in yeasts, revealing a likely role in fungal niche competition and establishing an entirely new, expansive family of aerolysin-like proteins.

**IMPORTANCE:**

Pore-forming toxins are potent biological weapons used across nature, from virulence factors to immune defense proteins. This study identifies K62, a little-known antifungal toxin produced by a wild yeast, as a structural and functional relative of the aerolysin family, which is well-known for forming damaging pores in cell membranes. Using structure prediction, molecular simulations, and biochemical analysis, we show that K62 assembles into large, stable pore-like complexes. Remarkably, K62 is just one member of a large and previously unrecognized family of similar toxin-like proteins found in fungi, plants, and bacteria, including pathogens that affect humans and crops. These findings uncover an unexpected evolutionary link across kingdoms, suggesting that pore-forming toxins may play a widespread role in fungal pathogenesis and microbial warfare. This work lays the foundation for understanding a new group of antifungal molecules and their potential impacts on health, agriculture, and microbial ecology.

## INTRODUCTION

Fungal killer toxins were first identified as being produced by the model yeast *Saccharomyces cerevisiae,* and since then, hundreds of other strains and species of killer yeasts have been isolated (1–5). Killer toxins represent an underutilized source of antifungals to combat the threat posed by pathogenic and spoilage fungi, despite the success of killer yeasts in inhibiting these fungi in laboratory studies and commercial trials (such as references 6–11). Given the hundreds of known killer yeasts, relatively few killer toxins have been studied empirically, and there are only five empirically determined tertiary structure models available to aid in undertanding killer toxin function. Notably, no structural models exist for killer toxins produced by *Saccharomyces* yeasts, despite a general mechanistic understanding of the *S. cerevisiae* killer toxins K1, K2, and K28 (12–14). Advances in protein structure prediction and molecular dynamics (MD) simulations enable biologists to create high-confidence tertiary structure models from primary protein sequences (15). This is particularly useful for uncharacterized proteins, such as killer toxins, that lack sequence homology to other well-studied proteins, as it enables alternative searches for structural homologs. The identification of conserved motifs and domains between killer toxins and proteins with solved empirical structures can be used to understand mechanisms of intoxication and drive the exploration of novel antifungals to fight disease and spoilage.

The unique antifungal activities of killer toxins have been frequently used to identify novel toxins in *Saccharomyces* yeasts. This is in addition to contemporary methods of nucleic acid sequencing, which have accelerated the identification of killer toxin genes that can be encoded by cytoplasmic double-stranded RNAs associated with mycoviruses (12, 16, 17). Of the 11 known *Saccharomyces* killer toxins (K1, K1L, K2, K21, K28, K45, K62, K74, Klus, KHR, and KHS), four have been recognized as being capable of attacking membranes as the primary means of killing cells. Specifically, K1 has been characterized as a pore-forming toxin as it causes the efflux of potassium ions from targeted cells (18). Other killer toxins (K1L, K2, and K21) have been presumed to be ionophores based on their ability to damage membranes, related secondary structures, or requirement for similar genes to intoxicate fungal cells (19–21). Recently, tertiary structure predictions and MD simulations of K2 have revealed striking homology to salt-mediated killer toxin (SMKT), which supports a mechanism of membrane attack and cell permeabilization by K2 (22, 23). K28 is the only other well-studied *Saccharomyces* killer toxin and arrests the fungal cell cycle by a mechanism involving retrograde transport from the cell surface to the nucleus (14). The killer toxin K62 is one of six *Saccharomyces* killer toxins that have not been well characterized and has a unique spectrum of antifungal activities against yeasts. It is also one of the only killer toxins that is unable to inhibit the growth of *Nakaseomyces glabratus* (6). The previous misclassification of K62 as K1, along with its uniqueness as a killer toxin, justified further investigation of its structure and function.

In this study, we characterized the antifungal properties of K62 from *Saccharomyces paradoxus* and investigated its monomeric and oligomeric structure using AlphaFold and MD simulations. This revealed that K62 is a structural homolog of aerolysin family toxins, which are beta-barrel pore-forming toxins that are widely distributed across bacteria, fungi, and animals (24, 25). These proteins are highly toxic to eukaryotic cells and are responsible for significant disease in ruminants (26–28). Aerolysin toxins have two domains: a highly variable N-terminal receptor-binding domain and a C-terminal aerolysin core domain, the latter of which is characterized by a conserved five beta-strand motif (25). The assembly of a beta-barrel pore by the C-terminal domain kills targeted cells by inducing uncontrolled ion leakage (29, 30). Consistent with the stable pore or pore-like structures assembled by aerolysin family toxins, K62 assembled heat- and detergent-resistant oligomers. This identified K62 as the first aerolysin-family toxin in a large and previously undescribed new family of toxin-like proteins widely distributed across fungi, plants, and bacteria.

## RESULTS

### The characterization of the K62 killer toxin from *S. paradoxus*

**Fig 1:**
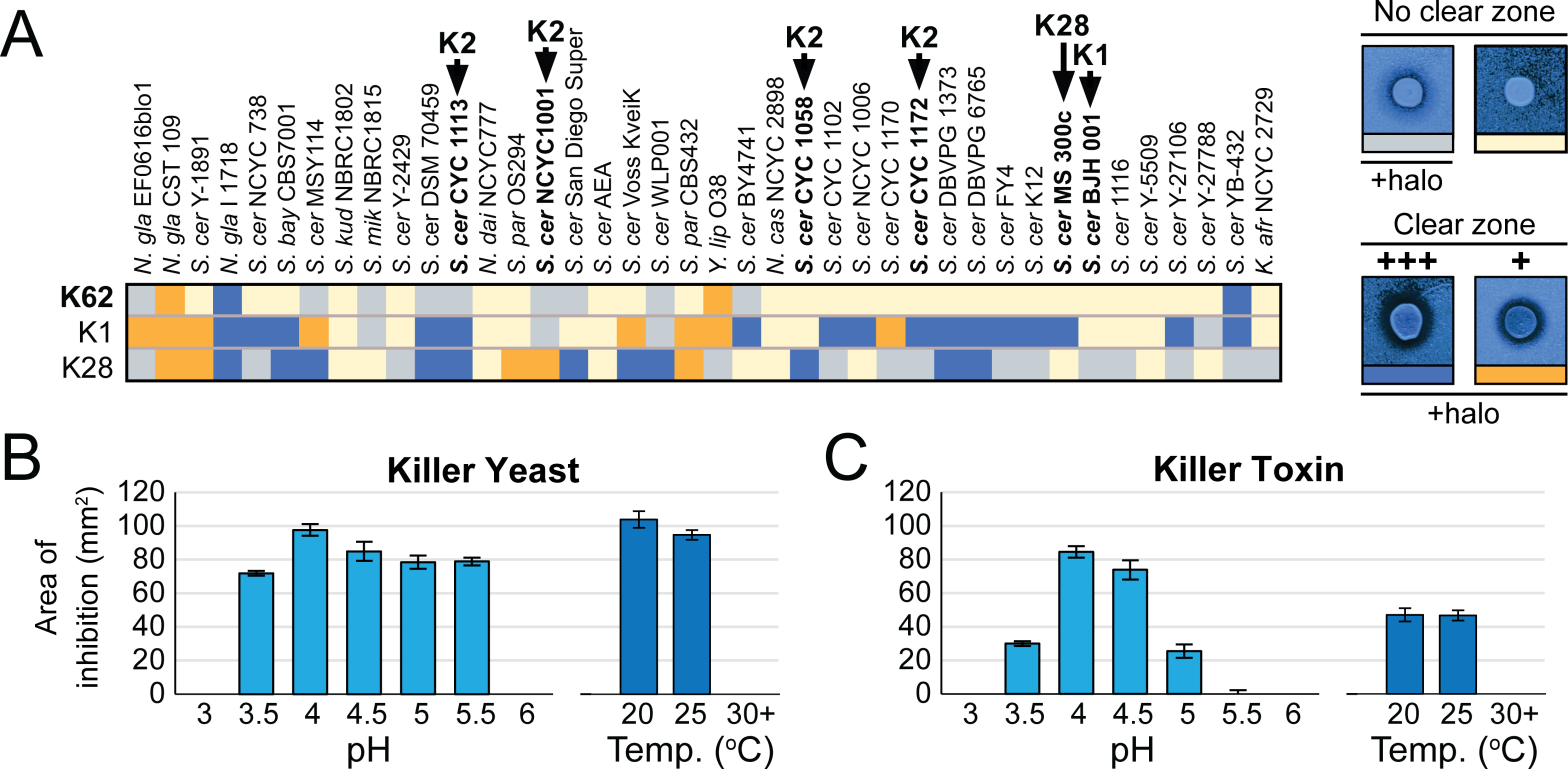
The antifungal activity of K62. (**A**) Qualitative spectrum of K1, K28, and K62 antifungal activities across 40 strains of susceptible yeasts as assayed on YPD agar with methylene blue. The darkest shade of blue indicates the most severe growth inhibition, with a large (+++) zone of growth inhibition (clear zone) with methylene blue staining. Orange represents a small (+) zone of growth inhibition with methylene blue staining. Gray indicates only methylene blue staining, and yellow indicates no observed antifungal effects. Killer toxin strains are marked with arrows and the toxins they produce. (**B**) Cells of the killer yeast *S. paradoxus* Q62.5 and (**C**) precipitated K62 killer toxin at 25°C across a pH range of 3 to 6 and at pH 4.6 at three temperatures.

To determine the antifungal activities of K62, 40 different strains of yeasts were challenged by *S. paradoxus* Q62.5 and other killer yeasts expressing the canonical killer toxins K1 or K28. Strains chosen to test K62 susceptibility included those that we had previously determined as being sensitive to known killer toxins, with the majority (82%) being *Saccharomyces* yeasts. Growth inhibition was qualitatively assessed by the killer plate assay, where killer yeasts are inoculated onto a lawn of potentially susceptible yeasts on YPD agar plates buffered to pH 4.6 with the redox indicator methylene blue. After incubation, zones of growth inhibition and methylene blue staining, indicating cell death, were observed if the lawn strain of yeast was susceptible to a killer toxin. Of the yeast strains tested for killer toxin susceptibility, 33% were susceptible to K62, 70% were susceptible to K1, and 75% were susceptible to K28 (Fig. 1A). In addition to fewer yeasts being sensitive to K62, halos of growth inhibition were qualitatively smaller than those formed by K1 and K28. K62 could not kill K28 or K1 killer yeasts but could inhibit the growth of two of four K2 killer yeasts (Fig. 1A). These data support previous studies, which show that K62 has a novel spectrum of antifungal activity compared with other canonical killer toxins. Antifungal activity of K62 was observed between pH 3.5 and 5.5 and was the highest at pH 4.0 for both the killer yeast (Fig. 1B) and K62 concentrated by ammonium sulfate precipitation (Fig. 1C). K62 was most active at 20°C and 25°C, with no activity at either 30°C or 37°C, which was broadly consistent with other previously characterized *Saccharomyces* killer toxins (Fig. 1B and C).

### K62 is encoded on a dsRNA satellite, and neither is required for K62 immunity

**Fig 2:**
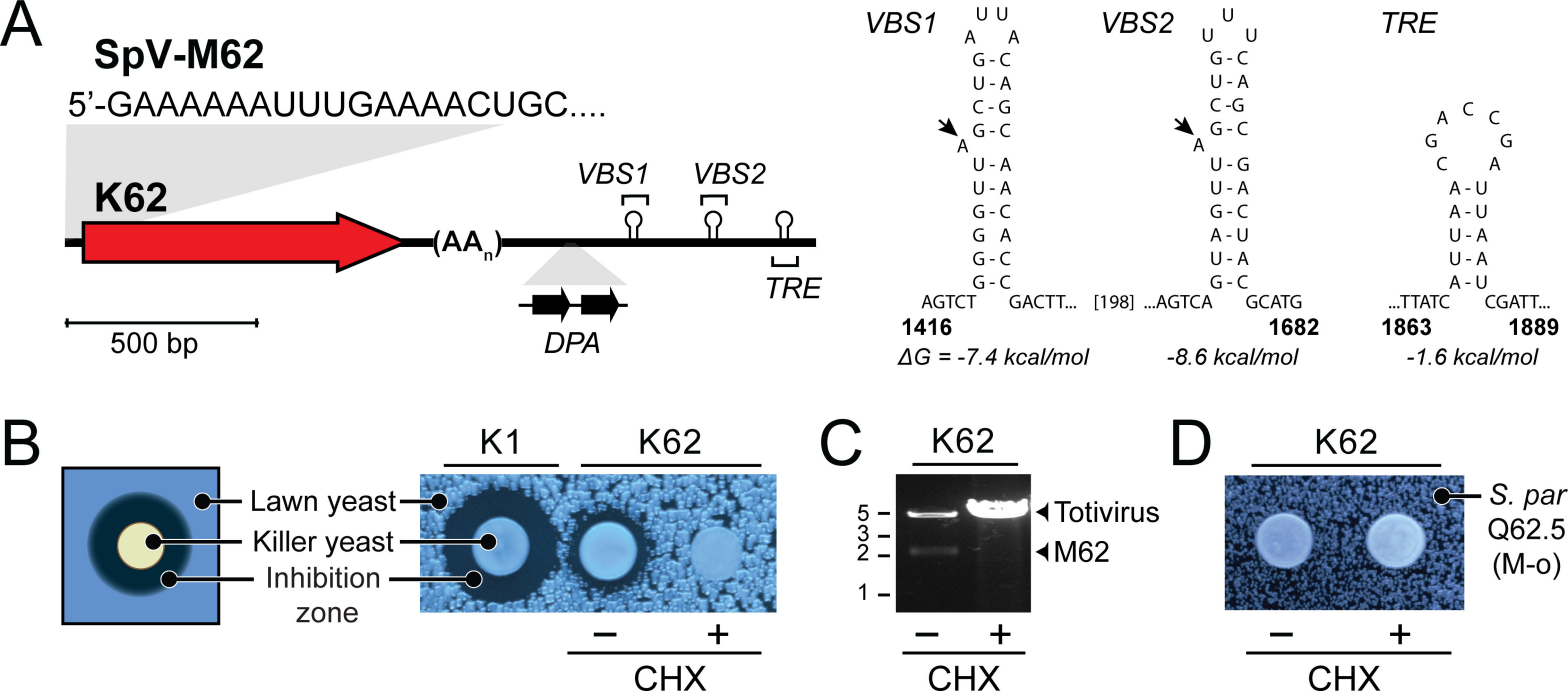
K62 is an antifungal toxin encoded on a dsRNA M satellite. (**A**) A schematic representing the M62 satellite dsRNA with the K62 ORF, polyadenine tract (AA_n_), two direct repeats of the *DPA* sequence, two putative viral binding sites (*VBS1* and *VBS2*), and a terminal recognition element (*TRE*). Stem-loop predictions of VBS1, VBS2, and TRE with ΔG for dissociation of the stem-loop. Arrows on stem loops mark adenine bulges characteristic of VBS sites. (**B**) Killer assay with *S. paradoxus* Q62.5 before (CHX−) and after (CHX+) cycloheximide treatment. A K1 killer yeast was used as a positive control in the killer assays. (**C**) Agarose gel electrophoresis analysis of dsRNA from *S. paradoxus* Q62.5 before (CHX−) and after (CHX+) cycloheximide treatment. (**D**) Killer assay with *S. paradoxus* Q62.5 before (CHX−) and after (CHX+) cycloheximide treatment using the cycloheximide-cured strain of OS169 lacking M62 (M62-O) as the lawn strain.

The production of killer toxins by *Saccharomyces* yeasts is frequently associated with double-stranded RNA (dsRNA) totiviruses from the family *Totiviridae* and toxin-encoding dsRNA “M” satellites (17). Totiviruses support the production of killer toxins by acting as helper viruses, enabling the replication and encapsidation of satellite dsRNAs (17). *S. paradoxus* Q62.5 was previously found to harbor a totivirus and an M satellite named M62. The designation of K62 as a novel toxin was confirmed after the genetic sequence of M62 was elucidated (31). M62 is 1,935 bp in length and organized similarly to other killer toxin dsRNA satellites (Fig. 2A). The K62 gene is positioned at the 5′ end of the dsRNA and separated from the 3′ untranslated region (UTR) by a central polyadenine tract. As with many other dsRNA M satellites, the 5′ terminus of M62 has a nucleotide sequence that begins with guanine, followed by a short polyadenine tract (GAAAAAA) and a 5′ UTR of 35 bp before the K62 gene. The 3′ UTR has two copies of the downstream of poly A (*DPA*) sequence (consensus 5′-CTCACCYTGAGNHTAACTGG-3′) and stem-loops indicative of a terminal recognition element (*TRE*), and two viral binding sites (*VBS*) (21). The *VBS* stem-loops have a characteristic adenine bulge, and both the *VBS* and *TRE* elements have low dissociation energies and are likely necessary for dsRNA replication and encapsidation (32). Together, these data confirm that the general organization of M62 is similar to that of other M satellite dsRNAs identified in yeasts.

To ensure that the killer phenotype of *S. paradoxus* Q62.5 was solely due to K62 expression from M62, the strain was cured of M62 (M62-o) by exposure to cycloheximide, resulting in the loss of the killer phenotype (Fig. 2B). Extraction of dsRNAs from *S. paradoxus* Q62.5 before and after curing demonstrated that the loss of M62 was correlated with the loss of killer toxin production (Fig. 2C). Curing of M62 did not cause the loss of the associated totivirus (~5 kb) but resulted in a large increase in totivirus copy number, likely due to the loss of M62 parasitism (Fig. 2C). These data confirm that M62 is structurally similar to other satellite dsRNAs and is solely responsible for the expression of K62 by *S. paradoxus*.

Killer yeasts have different mechanisms of immunity to protect from mature extracellular toxins. Self-protection can be supplied by the immature preprotoxin (K1 and K28), signal sequence (K2), or other genome-encoded factors (KP4 and zygocin). To determine whether K62 encodes self-immunity, M62-o strains were challenged by the wild-type K62-expressing killer yeast strain. Unlike other canonical *Saccharomyces* killer toxins, the loss of the M62 satellite did not result in a loss of immunity, suggesting that K62 immunity is genome-encoded (Fig. 2D; Fig. S1). Having established the genetic basis of K62-mediated killing and the absence of a K62-mediated immunity mechanism, the structural characteristics of the K62 toxin were investigated using molecular modeling.

### The killer toxin K62 is predicted to share structural homology with the aerolysin family toxins

**Fig 3:**
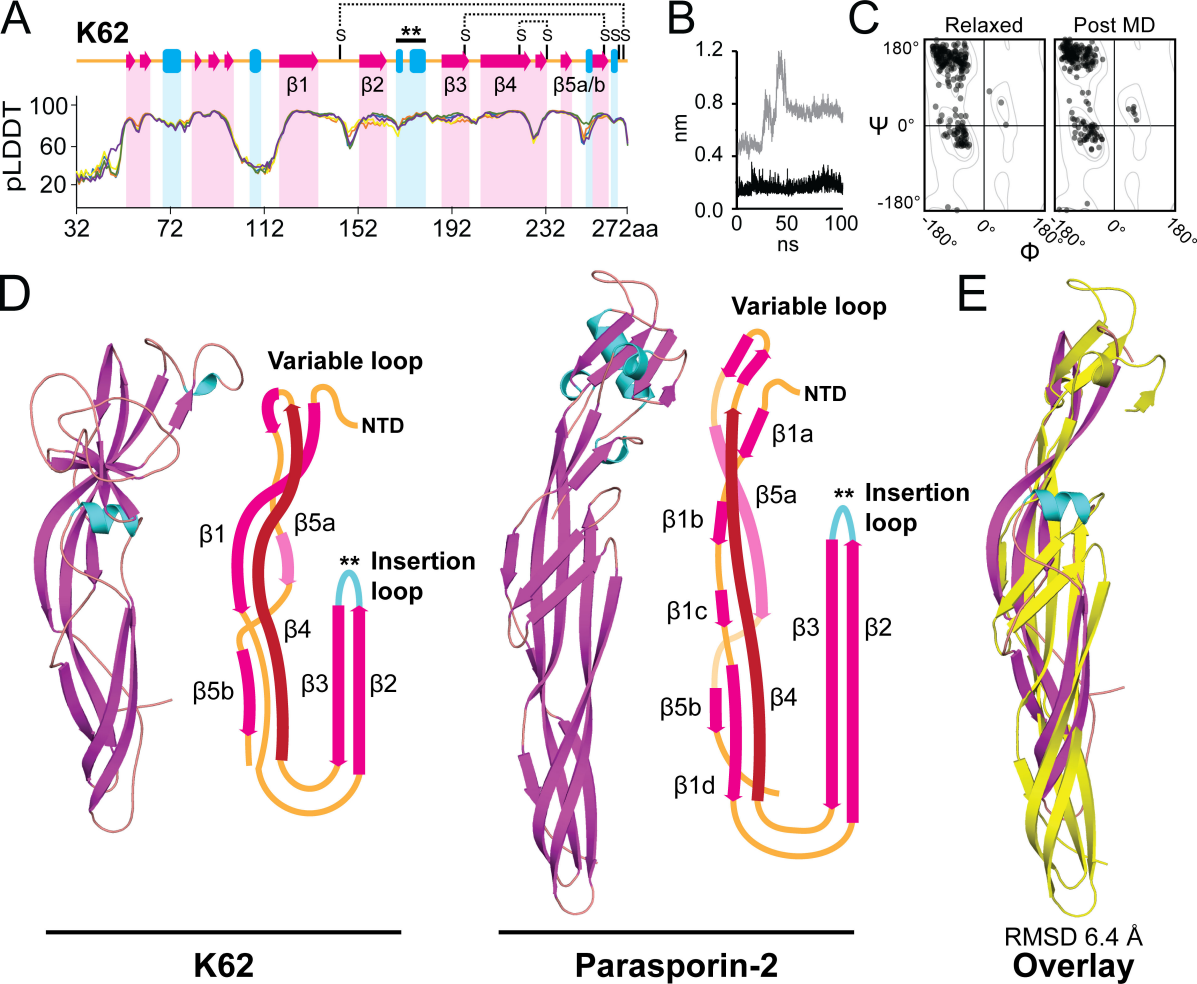
K62 is a structural homolog of aerolysin-family toxins. (**A**) Linear representation of the top K62 AlphaFold2 prediction relative to pLDDT (predicted local distance difference test) scores from five AlphaFold2 models. Alpha helices are represented as blue rectangles, beta strands as red arrows, and unstructured regions as orange lines. Predicted disulfide linkages are indicated as horizontal dashed lines. The insertion loop is marked by two asterisks. (**B**) K62 RMSD over a 100 ns GROMACS MD simulation. The gray line is all backbone atoms, and the black line is aerolysin core domain backbone atoms (residues 112–272). (**C**) Ramachandran plots of general residues (non-proline/glycine) generated using the SWISS structure assessment tool before and after performing MD simulation. (**D**) AlphaFold2 and molecular dynamics tertiary structure prediction of K62 and the crystal structure of parasporin-2 (PDB: 2ZTB). A schematic representation of the aerolysin core domain of K62 and parasporin-2 is presented to the right of each structure to highlight the organization of the secondary structure and the relative positioning of the variable and insertion loops. (**E**) Superposition of the aerolysin core domains of K62 (magenta) and parasporin-2 (yellow).

K62 is a 272 amino acid protein, similar in length to other *Saccharomyces* killer toxins (averaging 320 amino acids), but with no amino acid sequence homology. To gain insight into the molecular structure of K62, AlphaFold2 was used to generate tertiary structural models (15). The top-ranked K62 model had an average per-residue predicted local distance difference test (pLDDT) score of 81.4/100, with a maximum score of 97.4, indicating high confidence in the structure (Fig. 3A). Regions of high confidence were primarily aligned with alpha helices and beta strands, whereas lower confidence scores corresponded to disordered regions (Fig. 3A). The secondary structure of K62 had a large proportion of beta strands (41.6%) and fewer alpha helices (4.1%). This structure contrasted with other killer toxin crystal structures, with an average composition of 30.1% beta-strands and 20.9% alpha-helices (Table S1). In addition, the AlphaFold2 tertiary structure model of K62 predicted an elongated beta-sheet structure measuring ~86 × 40 Å that differed from the more globular structures of killer toxins. Due to the significant structural differences, K62 is likely to have a unique mode of antifungal action compared with all other previously described killer toxins.

AlphaFold2 prediction software creates static structural models. Therefore, it is important to use MD simulations to model the stability, biophysical properties, and interactions between atoms to generate more realistic tertiary protein structures. To assess structural stability and reduce clashes between atoms, a 100 ns MD simulation was performed using the AlphaFold2 predicted structure as an input. As expected for a predictive model, the root mean squared deviation (RMSD) began increasing within the first nanosecond of the simulation relative to the starting structure, indicating a movement away from the configuration of the original AlphaFold2 structure (Fig. 3B). RMSD peaked at 1.2 nm between 40 and 50 ns before stabilizing at 0.8 nm after 50 ns, indicating a structure that had reached a stable conformation (Fig. 3B). The MD simulation improved Ramachandran favored phi and psi angles of the protein to 95.0% and reduced outliers to 0.5% (Fig. 3C; Table S2). A comparison of the final K62 structure with the starting AlphaFold2 model revealed an RMSD of 0.72 nm over the entire protein and an RMSD of 0.19 nm when comparing only the conformation of the C-terminal domain (Fig. S2). RMSD of the C-terminal residues 112–272 was stable around 0.17 nm and never went above 0.36 nm (Fig. 3B). The stability of the C-terminal domain indicated that the N-terminal domain of K62 was responsible for the majority of flexibility during MD simulation. Overall, the predicted AlphaFold2 model of K62 remained stable during the later part of the MD simulation, supporting the adoption of a biologically relevant conformation.

To identify tertiary structure homologs of K62 in the protein database, the last frame of the 100 ns MD simulation of K62 was used to perform analysis by distance-matrix alignment (DALI) using pairwise structural alignment and a search of the protein database (33). This analysis yielded 13 protein structures with confident Z-scores above 3.0, with 10 identified as belonging to the pore-forming aerolysin family of toxins, with one from the mushroom *Laetiporus sulphureus* (Table S3). The top structural match was an aerolysin family toxin named parasporin-2 (PDB: 2ZTB), with a Z-score of 5.1 (Fig. 3D). The Phyre homology modeling server corroborated these findings, identifying a 70-residue region of K62 that matched parasporin-2 with 69.8% confidence (34). The K62 structural model exhibited a five beta-strand folding pattern characteristic of the conserved aerolysin core domain (Fig. 3D). Beta strands 1, 4, and 5 formed a stable structure, whereas strands 2 and 3 positioned a loop analogous to the pore-forming insertion loop of aerolysin toxins (Fig. 3D) (25). Based on its similarity to parasporin-2, the insertion loop of K62 was predicted to be 25 amino acids in length (residues 167–192) that alternated between hydrophobic and polar residues (Fig. S3). This region of K62 was enriched with serine and threonine residues, which aid in oligomerization and enhance flexibility during membrane interaction and pore formation in other aerolysins. The K62 model was overlaid with the crystal structure of the top DALI hit, parasporin-2, for structural comparisons (Fig. 3E). Confirming the structural similarity to K62, the C-terminal aerolysin domain of parasporin-2, which is responsible for pore formation, aligned with K62 residues 112–272. The RMSD of the overlaid structures was 6.39 Å, despite only 13.6% sequence similarity (27). In contrast, the K62 N-terminal domain (residues 31–112) was more dissimilar to parasporin-2, with an RMSD of 7.9 Å. No structural homologs of the K62 N-terminal domain were identified by DALI in the protein database. The structural similarities of K62 to aerolysin family toxins motivated further investigation of a possible pore-forming mechanism of this killer toxin.

### K62 from *S. paradoxus* is active when expressed by *S. cerevisiae* and forms heat-stable oligomers

**Fig 4:**
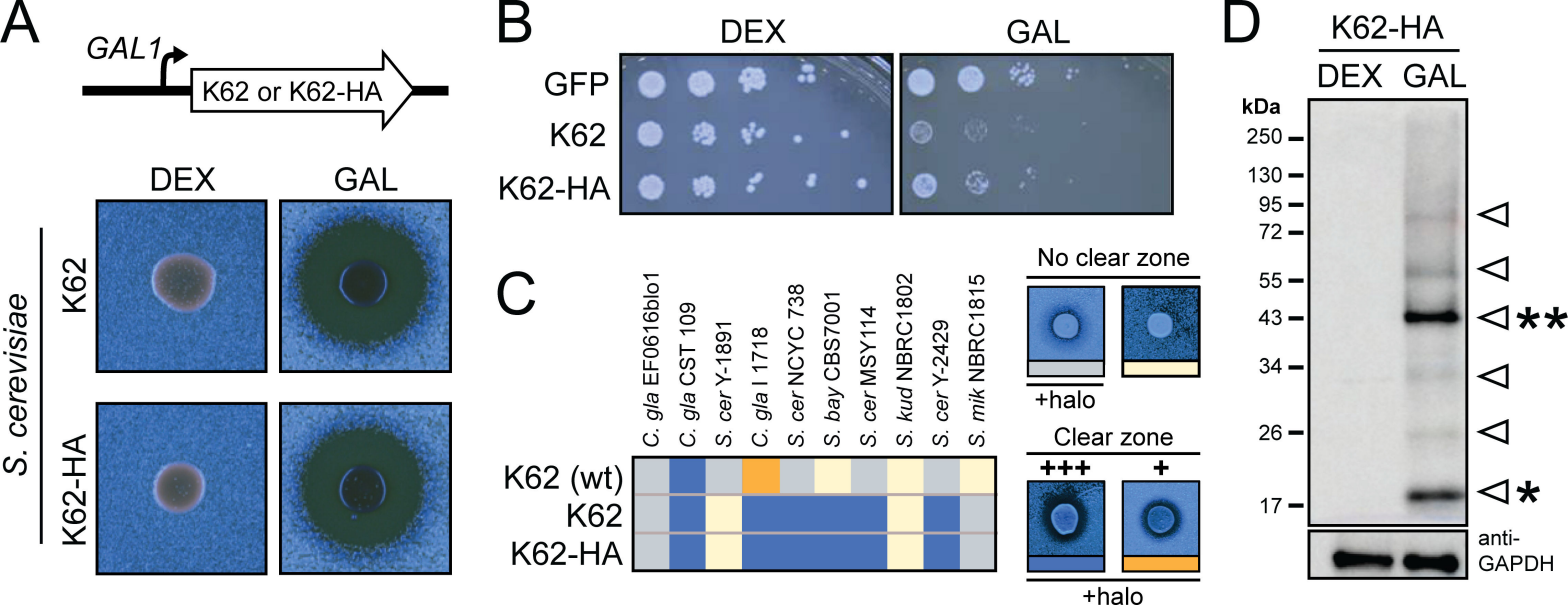
Ectopic expression of K62 by *S. cerevisiae* induces the killer phenotype. (**A**) (Top) A galactose-inducible promoter controlled K62 expression. (Bottom) Killer assay demonstrating galactose-dependent induction of genome-integrated copies of K62 and K62-HA by laboratory strain *S. cerevisiae* CRY1; (**B**) 10-fold dilutions of *S. cerevisiae* CRY1 were spotted to assay cell health with and without K62 and K62-HA expression from a high-copy plasmid. (**C**) The representative antifungal activity of K62 expressed by *S. paradoxus* [K62 (wt)] compared with galactose-induced expression of K62 or K62-HA by *S. cerevisiae*. The coloring and representative images of phenotypes correspond to different antifungal phenotypes when assayed using killer assay agar, as presented in Fig. 1 (*n* = 3). (**D**) Western blot of *S. cerevisiae* whole cell lysate from cells expressing (GAL) or not expressing (DEX) K62-HA. The protein was analyzed by denaturing SDS-PAGE and probed with an anti-HA HRP-conjugated primary antibody. GAPDH serves as a protein loading control. Arrowheads represent different oligomeric states of K62 with * and ** representing predicted monomeric and dimeric K62.

To confirm that K62 is solely responsible for the antifungal activities of *S. paradoxus* Q62.5, the K62 gene was expressed in laboratory strains of *S. cerevisiae* under the control of a galactose-inducible promoter. Zones of growth inhibition from K62 expression were only observed on galactose-containing plates, confirming that the expression of K62 is sufficient to induce the killer phenotype (Fig. 4A). However, the induction of K62 caused methylene blue staining of *S. cerevisiae* strain CRY1, indicating killer-toxin-associated cell death. Spot dilution assays confirmed that high copy overexpression of K62 is toxic to *S. cerevisiae,* and strain BY4741 was more sensitive to K62 than CRY1 (Fig. 4B; Fig. S4). The toxicity of K62 appeared reduced when overexpressed from a single-copy gene using strain CRY1 while still producing large zones of growth inhibition (Fig. 4A; Fig. S4). Recombinant K62 expressed by *S. cerevisiae* inhibited 87% of the yeasts that were sensitive to K62 expressed by *S. paradoxus*. The expression of K62 by *S. cerevisiae* also revealed a broader spectrum of antifungal activity, inhibiting two additional strains of yeast that had previously appeared resistant to native K62. The increased toxicity of K62 could have been due to its higher expression by *S. cerevisiae* relative to that of the M62 dsRNA satellite (Fig. 4C). The inhibition of *S. cerevisiae* Y-1891 by K62 expressed by *S. paradoxus* and not by ectopically expressed K62 might indicate that there are additional killer toxins or antifungal molecules that *S. paradoxus* produce in addition to K62 (including a genome-encoded K62 homolog, as discussed in a later section).

Aerolysin family proteins have been shown to exist as dimers and high molecular weight oligomers in solution. To detect K62 by western blotting, a hemagglutinin (HA) epitope tag was added to the C-terminus of the toxin (K62-HA). Adding the tag only slightly reduced the antifungal activities and cytotoxicity of K62 (Fig. 4A and B). Induction of K62-HA enabled the detection of an apparent monomer above 17 kDa that was smaller than predicted by the primary sequence (~28 kDa) (Fig. 4D). A second protein was detected by western blotting at ~43 kDa, consistent with a dimer of K62-HA. Importantly, this dimer was resistant to denaturation, as it was stable after incubation at 98°C with a detergent (SDS) and reducing agent (beta-mercaptoethanol) (Fig. 4D). A further attempt to dissociate the dimer by boiling with 4 M guanidine hydrochloride was also unsuccessful (Fig. S5). These data are consistent with those of other aerolysin family proteins, which form stable dimers in solution. Other distinct minor protein species were also observed migrating above and below the putative dimer, with some high molecular weight protein smears above the 250 kDa marker (Fig. 4D). The same pattern of K62-HA monomers and oligomers was also observed in cell-free culture media, confirming that K62 was exported from the cell (Fig. S5). The detection of high molecular weight complexes of K62 suggested oligomerization in solution, which was consistent with the behavior of aerolysin family toxins that assemble oligomeric pre-pores and pores in solution.

**Fig 5:**
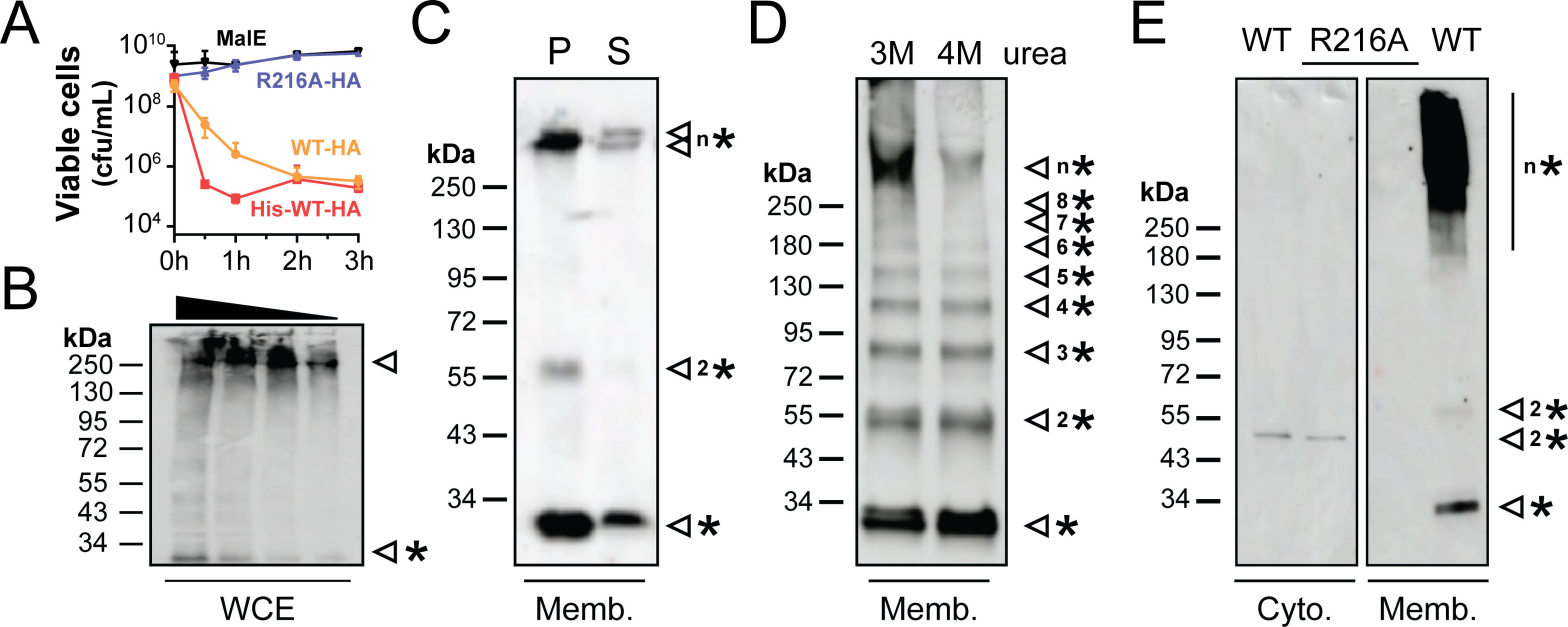
The expression of K62 is lethal to *E. coli* and enables the assembly of heat-stable, high molecular weight membrane-associated oligomers. (**A**) The viability of *E. coli* was measured for 3 h by colony-forming units (cfu) after induction (time 0) of K62 with different mutations and epitope tags compared with the expression of maltose-binding protein (MalE) (*n* = 3). Error bars are the standard deviation. All K62 proteins (wild-type [WT] and the R216A mutant) encode a C-terminal hemagglutinin tag (HA). His-WT-HA also encodes a hexa-histidine N-terminal tag. (**B**) Western blot analysis of *E. coli* whole cell extracts (WCE) after induction of K62-HA. (**C**) Western blot analysis of the insoluble pellet “P” or SDS solubilized “S” fractions of purified membranes extracted from *E. coli* cells expressing His-K62-HA (Memb.). (**D**) Western blot analysis of urea and SDS-treated *E. coli* membrane fractions (Memb.) after induction. Arrows represent His-K62-HA oligomers with differences in their apparent molecular weights. Asterisks indicate the most likely oligomeric state of His-K62-HA, where possible. (**E**) Western blot analysis of the cytoplasmic (Cyto.) and membrane fractions (Memb.) of *E. coli* expressing K62-HA (WT) and the K62(R216A) mutant (20 μg of protein loaded in each well). Arrows with one asterisk indicate the monomer, and the addition of a number indicates monomers (*), dimers (2*), trimers (3*), tetramers (4*), etc., of K62. N* was used when the oligomeric state could not be predicted.

To increase the yield of recombinant K62-HA for the investigation of oligomerization, the bacterium *Escherichia coli* was used to express the toxin without a eukaryotic N-terminal signal sequence. Upon induction of K62-HA with and without a polyhistidine tag, the OD_600_ of the *E. coli* culture plateaued, and cell viability decreased rapidly by up to three orders of magnitude (Fig. 5A). This rapid loss of cell viability was consistent with the toxicity of K62 observed upon its expression in *S. cerevisiae,* suggesting that K62 is capable of attacking bacterial membranes. Denaturing SDS-PAGE and western blot analysis of *E. coli* whole cell lysates harvested after induction of K62-HA confirmed the expression of a ~28 kDa K62 monomer that was larger than the monomer observed during expression in *S. cerevisiae*. This 28 kDa monomer was consistent with the molecular weight of K62 predicted by the primary sequence. However, most of the K62-HA protein migrated with an apparent molecular weight greater than 250 kDa, which was stable after boiling in SDS and reducing agent (Fig. 5B).

Since *E. coli* expressing K62 lost viability and aerolysin family proteins formed membrane pores, cell membranes were harvested after induction to look for the presence of oligomers. Western blotting of membrane fractions showed enrichment of K62-HA monomers (~28 kDa) and oligomers (>250 kDa) (Fig. 5C). K62-HA could only be effectively extracted and solubilized from the membrane fraction by incubation with 0.5% SDS, despite attempts to solubilize it with other detergents (Fig. 5C; Fig. S6). To confirm the solubility and large molecular weight of K62-HA, size exclusion chromatography of the SDS-solubilized fraction found that K62-HA eluted close to, but separate from, the void volume, indicating an apparent molecular weight of more than 600 kDa but less than 2,000 kDa (Fig. S7). To determine the oligomeric state of K62 oligomers, membrane fractions were treated with urea before boiling with gel loading buffer and analyzed by denaturing SDS-PAGE. This treatment successfully disassembled the quaternary structure of the K62 high molecular weight complex into discrete oligomers (Fig. 5D). Based on these data, the putative high molecular weight oligomer of K62 is at least nonameric based on observing octamers below a single larger complex of unknown composition (Fig. 5D). Together, these data suggest that like other previously characterized aerolysin family toxins, the large complex formed by K62-HA is likely a membrane-associated oligomeric pore or pore-like structure.

**Fig 6:**
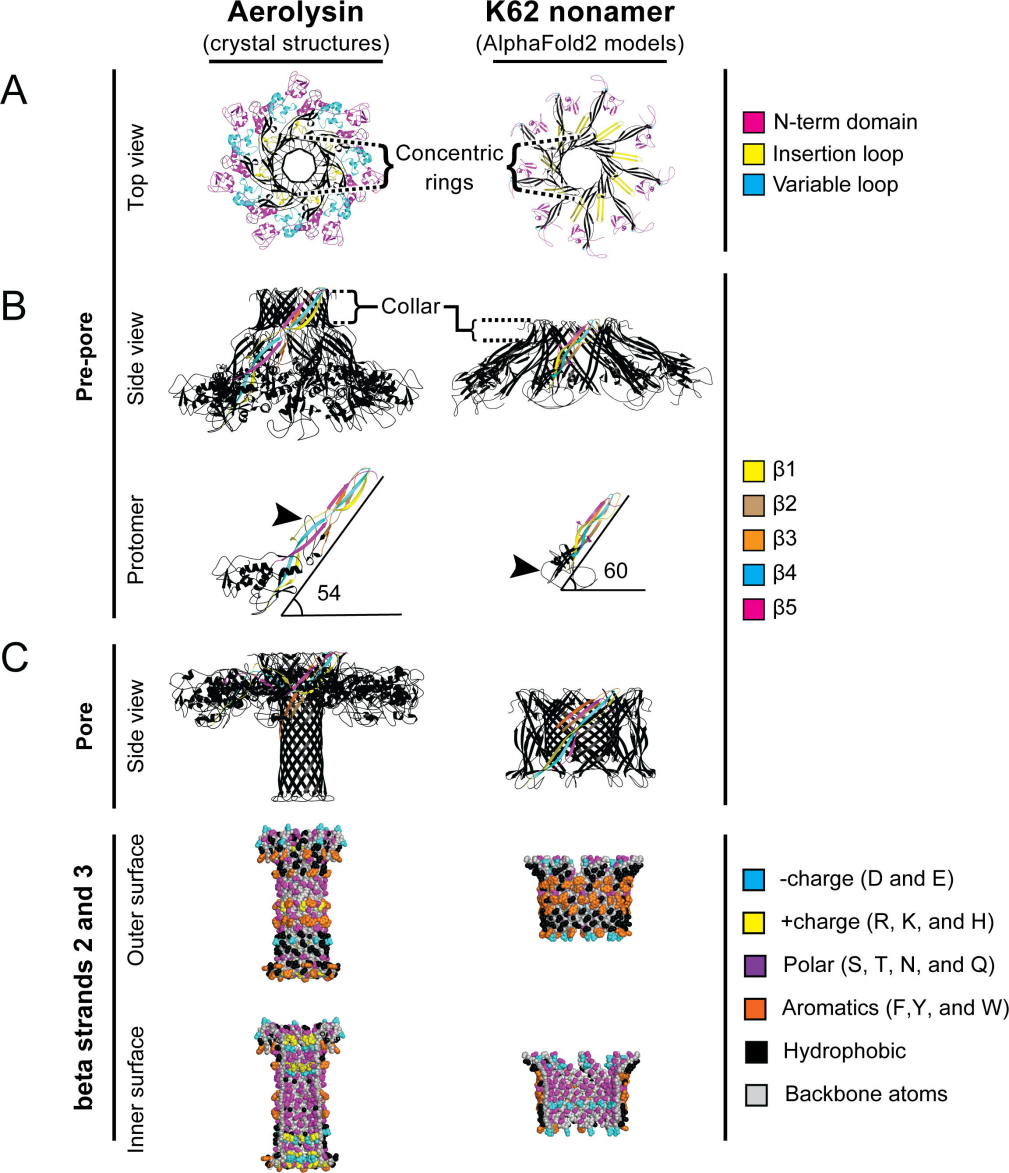
Comparison between the crystal structures of the aerolysin heptamer and the AlphaFold2 predicted K62 nonamer. (**A**) The top-down view is colored by functional regions of Aerolysin pre-pore structure 2JZH and the AlphaFold2 model of K62 nonamer lacking a signal sequence. (**B**) Side view colored by sheets. (Top) Aerolysin pre-pore structure 2JZH and AlphaFold2 model of K62 lacking signal sequence nonamer. (Bottom) A single subunit was extracted from pre-pore structures to highlight a similar organization of beta sheets and the insertion loop (arrowhead). (**C**) (Top) Aerolysin pore structure 2ZTB and AlphaFold2 model of K62 aerolysin core domain (amino acids 112-272) nonamer. Properties of the beta-barrel of the pore conformation are depicted as spheres and colored by residue properties. Aerolysin beta barrels are formed by the insertion loop and beta strands 2 and 3. (Middle) The outer surface of pores. (Bottom) Cutaway of the pore formed by beta strands 2 and 3 to view the inner pore surface.

The mutational analysis of bacterial aerolysins has identified residues essential for oligomerization. Using the K62 structural model and biochemical similarity to a residue in aerolysin that is essential for oligomerization, the amino acid R216 in K62 was predicted to play a role in oligomerization (35). The expression of the mutant K62 R216A-HA was not lethal to *E. coli*, indicating that this was a loss-of-function mutation (Fig. 5A). There were similar amounts of K62 in the wild type and R216A mutant soluble cell lysates, but membrane fractions of *E. coli* expressing the K62-HA(R216A) did not contain high molecular weight complexes (Fig. 5E). Therefore, R216A appeared to prevent membrane interaction and the formation of high molecular weight oligomers by K62.

### Modeling the quaternary structure of K62

Since the expression of K62 in yeast and bacteria formed oligomers, AlphaFold2 was used to predict K62 oligomerization. Models were based on nonamers, informed by the migration patterns of K62 oligomers in SDS-PAGE gels and similarities to other aerolysin oligomers. The predicted K62 nonamer formed a ring-like structure similar to the aerolysin pre-pore (Fig. 6A; Table S4). Consistent with the aerolysin pre-pore, the N-terminal domain of K62 nonamer formed the bottom edge of the oligomer that would be predicted to interact with a target membrane before pore formation. Beta strands 2 and 3 of the K62 aerolysin core domain form an inner ring, whereas beta strands 1, 4, and 5 form the outer of two concentric rings, which are the same strands responsible for the similar concentric ring structure in the aerolysin pre-pore. The angle between the subunit and membrane of the predicted K62 nonamer is 60°, comparable with the 54° angle of the aerolysin pre-pore. The predicted insertion loop of K62 is oriented downward and toward the membrane, which would facilitate pore formation in the membrane (Fig. 6B).

Oligomer predictions for the isolated K62 aerolysin core domain (R112 to the C-terminus) revealed a strikingly different quaternary structure from the predicted K62 pre-pore-like oligomers. This model assembles the insertion loop into an amphipathic beta-barrel, similar to those found in aerolysin and epsilon toxin pores (Fig. 6C). The overall dimensions of the K62 beta-barrel resemble the pseudo-pore oligomer of aerolysin, which is shorter and wider than the mature pores of aerolysin and epsilon toxins (Fig. S8; Table S5). The inner surface of the K62 beta-barrel was lined with polar residues, likely facilitating the movement of water and charged ions. A ring of aspartic acid residues (D171) suggested potential cation selectivity, similar to other pore-forming proteins that use rings of charged residues to impart ion-specificity. The outer surface of the modeled K62 beta-barrel had rings of aromatic and hydrophobic residues that would be positioned to interact with the lipid core and phospholipid head groups of a membrane bilayer, like other aerolysins.

Unlike parasporin-2 and other aerolysin family proteins, which lack disulfide bonds in their core domains, K62 was predicted to form three disulfide bonds: one linking a flexible region between beta-strands 1 and 2 to the C-terminus (C146–C271), another stabilizing beta-strands 3 and 5b (C201–C262), and a third spanning a hairpin loop at the end of beta-strand 4 (C227–C237) (Fig. S9). Disulfide bonds in the predicted K62 nonamer were in the same configuration as in the monomer. Although aerolysin lacks the disulfide bonds present in K62, the corresponding regions in aerolysin also remained in close proximity in the monomer, pre-pore, and pore structures (Table S6), suggesting that the predicted disulfide bonds in K62 would not constrain conformational changes during oligomerization and pore formation. However, the mutation of cysteines to alanine in K62 resulted in non-functional K62 (Fig. S9). *In silico* mutagenesis indicated that C146A, C201A, C262A, and C271A would destabilize folding both of the K62 monomer and (except C269A) the oligomer (ΔΔG^folding^ > 2 kcal/mol) (Table S7). These data suggest that disulfide bonds maintain K62 stability, as shown for N-terminal domain disulfides of aerolysin (36).

### K62 aerolysin-like proteins are found in fungi, bacteria, and plants

**Fig 7:**
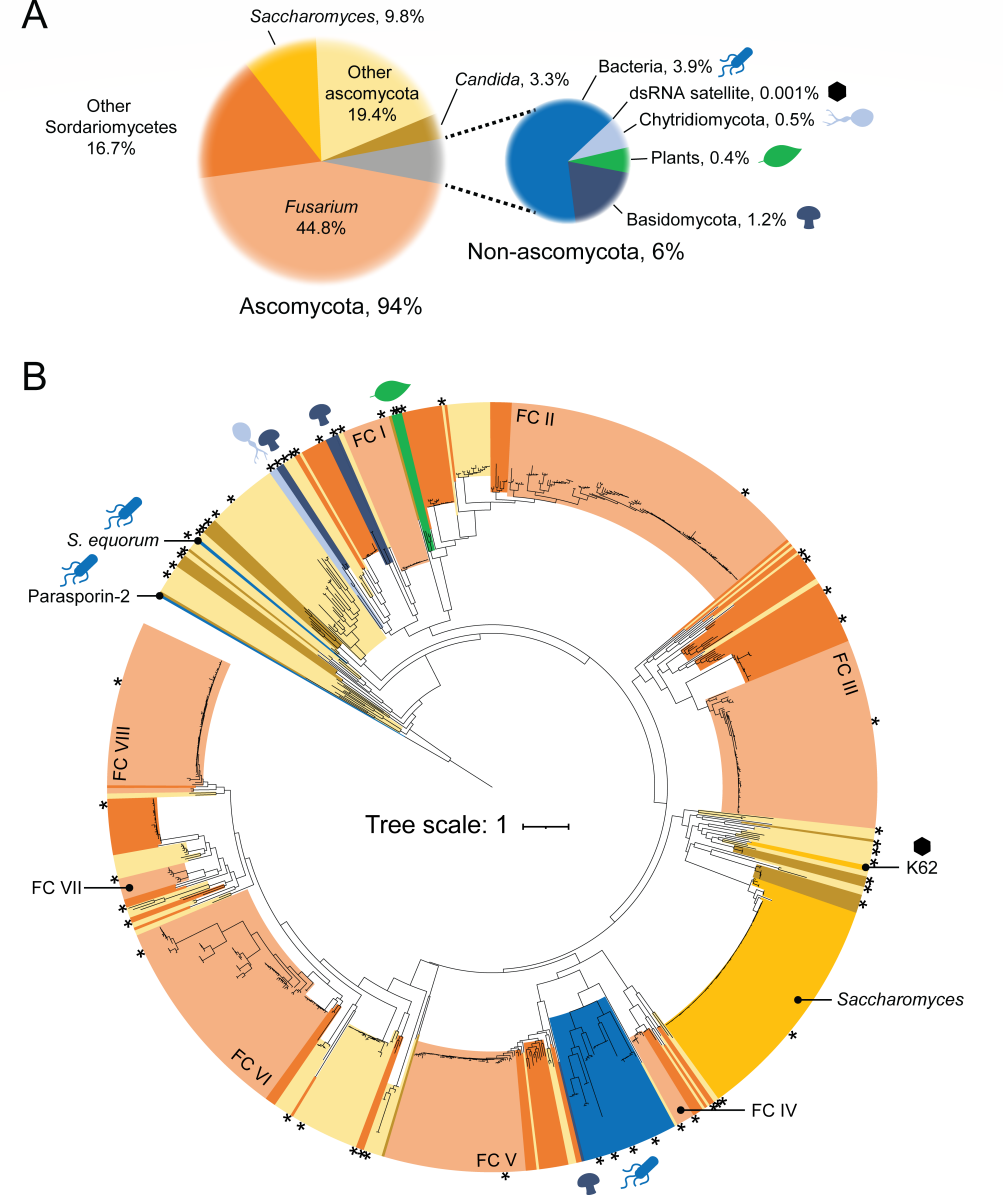
K62-like aerolysin proteins are found in fungi, bacteria, and plants. (**A**) Pie chart depicting the proportions of K62 homologs identified across fungi, bacteria, and plants. (**B**) A rooted phylogenetic tree colored the same as panel **A** shows a maximum likelihood model based on amino acid sequences of K62 homologs, calculated with 10,000 bootstrap iterations. Amino acid substitution models were determined according to the Bayesian information criterion, and it was found that the VT + R4 model was the best fit for the assembled data. The tree was rooted using the sequence of parasporin-2. Asterisks mark accessions used to predict tertiary structures. *Fusarium* clades are labeled as “FC” with numerals.

K62 has little to no primary sequence identity to known killer toxins or aerolysin toxins and has not been identified by previous bioinformatic screens aimed at discovering novel aerolysins (24, 25). To determine whether K62 is part of a larger family of toxins, PSI-BLAST was used to identify proteins with similar amino acid sequence and length (Fig. S10). Of the top 1,000 K62-like proteins (K62Ls) in fungi, bacteria, and plants, 99.8% were annotated as hypothetical or uncharacterized, and none were annotated as aerolysins. K62 was the only example of a dsRNA-encoded toxin; all of the other K62Ls appeared to be genome-encoded. Fungi of the Ascomycota represented 94.0% of the K62Ls, with the Basidiomycota and Chytridiomycota representing 1.2% and 0.5%, respectively (Fig. 7A). Within the Ascomycota, *Saccharomyces* yeasts compose 9.8% of all identified K62Ls, and 61.5% of K62Ls were encoded by the Sordariomycetes*,* with the majority (44.8%) from the *Fusarium* genus, including plant pathogens such as *Fusarium oxysporum* (12.2%) and *Fusarium graminearum* (0.7%) (Fig. 7A). Opportunistic human pathogens of the *Candida* and *Nakaseomyces* genera comprised 3.3% of the K62Ls identified and included *N. glabratus*, *C. parapsilosis*, and *C. auris*. Other opportunistic human pathogens included *Talaromyces marneffei*, *Emergomyces africanus*, *Blastomyces spp*., *Histoplasma capsulatum*, and *Paracoccidioides brasiliensis*. There were 39 bacterial K62Ls, of which 38 were from the class *Bacilli,* including the foodborne pathogen *Bacillus cereus*. Surprisingly, 0.4% of the K62Ls were identified in the genomes of seedless vascular plants (fern and clubmoss). Molecular modeling of a subset of K62 sequence homologs revealed that all had tertiary structures similar to K62 and aerolysin family toxins with high confidence (76% > 70 global pLDDT; 75% < 5 Å RMSD) (Fig. 7B; Table S1). For example, five tertiary structures predicted for K62Ls from representative bacteria aligned closely to K62, with the five beta-strand core domains defining them as aerolysin family proteins (Fig. S11; Table S8). In addition to tertiary structure conservation, cysteine residues critical for K62 function were also conserved in 22%–81% of the homologs, depending on the residue (Fig. S9). This analysis revealed that K62 represents one of the many hundreds of aerolysin homologs found in a diverse range of organisms, with the potential to be toxins.

To compare the relatedness of K62Ls, 883 sequences that were similar in length to K62 were aligned, and a phylogenetic model was constructed using a maximum likelihood methodology (Fig. 7B). The tree topology showed phylogenetic discordance with bacteria and plant K62Ls nested within the fungal K62Ls. This evolutionary pattern indicates the horizontal transfer of K62Ls from fungi to other, more distantly related species. K62 itself was part of a clade of yeasts from the *Saccharomycetes,* suggesting that it was mobilized onto a dsRNA from a yeast genome. To further describe the evolutionary history and dynamics of the K62L genes, several examples will be highlighted to demonstrate instances of horizontal and vertical gene transfer between organisms. For example, K62L genes are present in ancient seedless vascular plants, including ferns and clubmosses, and were the only higher eukaryotes to encode K62L genes. The phylogenetic positioning of the plant K62Ls nested within fungi of the Ascomycota suggests that these genes were acquired from fungi. The apparent absence of K62L genes from seed-bearing vascular plants (e.g., angiosperms and gymnosperms) and other more closely related primitive plants means that the common ancestor of plants did not have K62 or that it was lost from all lineages except those identified in the current study. The acquisition of K62-like proteins by these plants raises interesting questions about their function and whether they retain any properties common to the aerolysin family.

**Fig 8:**
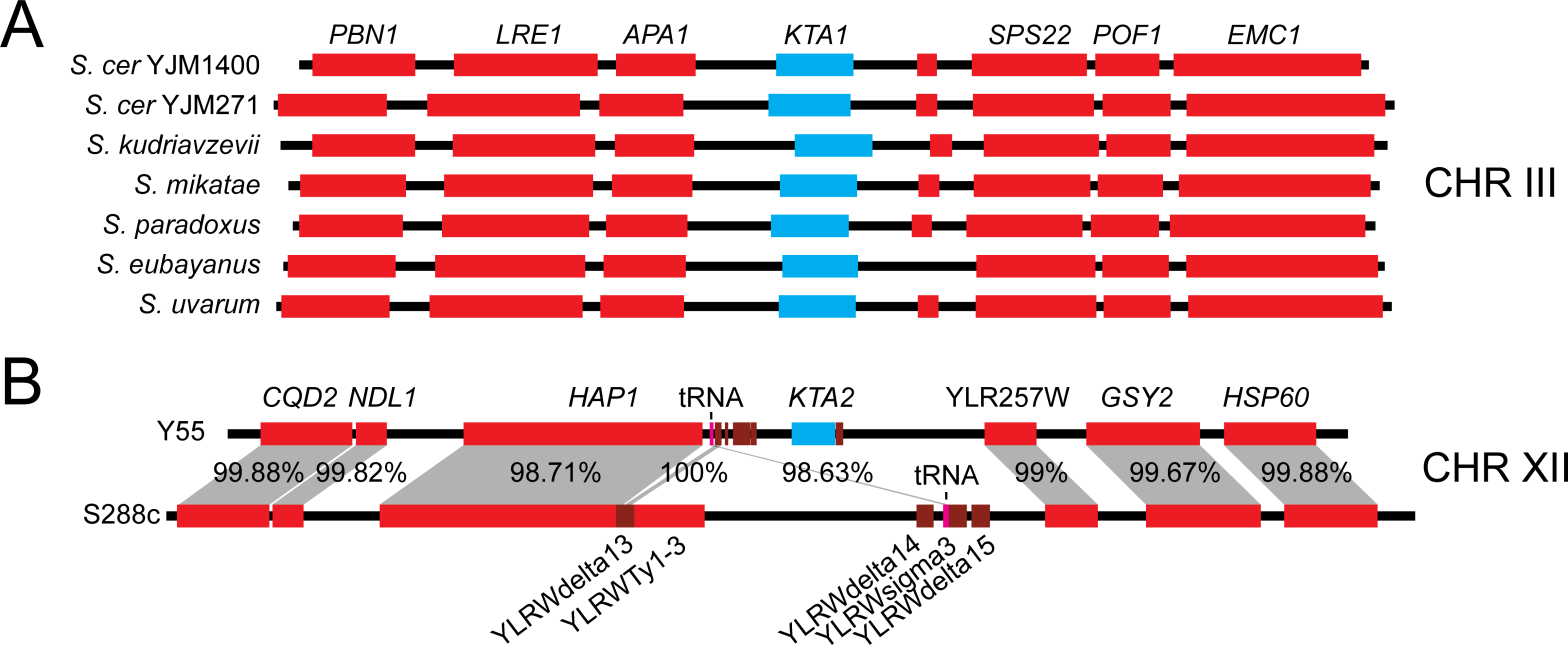
Vertical and horizontal gene transfer of K62 homologs in the *Saccharomyces* genus. (**A**) Synteny of *KTA1* across the *Saccharomyces* genus in chromosome III. (**B**) Synteny of chromosome XII of *S. cerevisiae* strains Y55 and S288c, indicating the unique *KTA2* insertion downstream of *APA1*. Red boxes represent genes, magenta indicates tRNA-ALA, brown denotes LTRs, and cyan *KTA1* or *KTA2*. Percentages represent the nucleotide conservation between genes and sequences of strains Y55 and S288c.

K62 was originally discovered as an M dsRNA-encoded killer toxin in the *Saccharomyces* yeast *S. paradoxus*, but no other homologs were identified on dsRNAs. Instead, K62Ls were found to be encoded on the genomes of all species of the *Saccharomyces* genus, with most (88.5%) originating from different strains of *S. cerevisiae* (Fig. 8A; Fig. S12). The *S. cerevisiae* homologs were identified as the uncharacterized gene YCL049C, which was provisionally designated *KTA1* for “killer toxin aerolysin 1.” *The KTA1* gene was syntenic across all *Saccharomyces* yeasts in chromosome III, including *S. paradoxus*, which harbors M62 (Fig. 8B). The phylogenetic arrangement of *KTA1* correlates with the phylogenetic relationship of *Saccharomyces* species, indicating the gene’s conservation over evolutionary time and presence in the last common ancestor of the genus (Fig. S12). There was no evidence of gene duplication of *KTA1* in *Saccharomyces* yeasts. However, there was a single instance of horizontal gene transfer of a K62L gene into chromosome XII of *S. cerevisiae* Y55, which was absent from all other *Saccharomyces* strains and species and is designated as *KTA2* (Fig. 8; Fig. S12). *KTA2* has 43.0% identity to K62L from the yeast *Tetrapisispora phaffii,* but only 20.2% identity to *KTA1* on the *S. cerevisiae* chromosome III. *KTA2* was inserted downstream (1,459 bp) from a tRNA gene and is flanked by long terminal repeats (LTRs), suggesting acquisition by retrotransposition. The absence of *KTA2* from the syntenic position in other *Saccharomyces* yeasts, including other strains of *S. cerevisiae*, suggested that it was acquired recently by horizontal gene transfer.

Most fungi that encode K62L belong to the *Fusarium* genus, a group of plant-pathogenic ascomycetes that can also cause opportunistic and sometimes life-threatening infections in humans. As was observed for the K62Ls from *S. cerevisiae*, the abundance of sequences from this genus likely reflects the large number of sequenced genomes available. *Fusarium* K62Ls are found in many monophyletic clades spread across the phylogeny, with fungi of the Sordariomycetes and other Ascomycetes being basal to these clades (Fig. 7B). However, the evolutionary history of K62L does not appear to accurately reflect the complete evolutionary history of the Sordariomycetes. In most cases, K62L ancestry is inconsistent across the clades and limited to only relatively few Sordariomycetes species, considering the diversity of the class (Fig. 7B; Fig. S13). Moreover, in several cases, there are long branches between *Fusarium* K62Ls and their nearest sister taxa (e.g., *Fusarium* clades I, III, VII, and VIII). Even within the *Fusarium* clades, the relatedness of K62Ls identified in the same or different species complexes appears discordant with the established phylogenetic models of the genus (37). For example, in *Fusarium* clade III, K62Ls in *F. redolens* (complex *redolens*), *F. gaditjirri* (complex *nisikadoi*), and *F. odoratissimum* (complex *oxysporum*) are nested within K62Ls from *F. oxysporum* (Fig. S13). Another example of phylogenetic discordance is observed in *Fusarium* clade I, with K62Ls from *F. commune* (complex *nisikadoi*)*, F. torulosum* (complex *tricinctum*), and *F. odoratissimum* (complex *oxysporum*) being >98% identical to K62Ls from *F. oxysporum* and separated by short branch lengths that do not accurately reflect the genetic distance between these species (Fig. S13; Table S10). These examples illustrate the complex evolutionary histories of K62Ls in the *Fusarium* genus, suggesting horizontal transfer between the fungi of the Sordariomycetes and other ascomycetes.

K62Ls are predominantly found in fungi, but with a small subset in bacteria; the majority are in a single clade of gram-positive bacteria dominated by the order Bacilli. These bacterial K62Ls are a sister taxon with Sordariomycetes fungi, suggesting that bacteria have acquired these toxins from fungi (Fig. 7B). The closest fungal K62L to this bacterial clade is a hypothetical protein from *Viridothelium virens*, a lichen-forming Dothideomycete, with 20% identity to a K62L from the most closely related *B. cereus* strains (Fig. S14). Within this bacterial clade, there is discordance between the K62L phylogeny and the established bacterial phylogeny (Fig. S14). For example, K62L from *Intestinibacter bartlettii* of the class Clostridia is nested within K62Ls from the class Bacilli. Even in the K62Ls from the class Bacilli, *Baia soyae* of the bacterial family *Thermoactinomycetaceae* is nested with K62Ls of the family *Bacillaceae* instead of being an outgroup (38). There are also two distinct lineages of K62L in *Bacillus cereus,* indicating that K62Ls have diverged since their acquisition from fungi. The horizontal transfer of the bacterial K62L genes is likely facilitated by their presence on plasmids in *B. cereus* and *B. thuringiensis,* in addition to genomic K62Ls (Fig. S14; Table S11) (39). The high nucleotide and amino acid identity of genomic versus plasmid-encoded sequences (97% nucleotide identity) suggests that K62L has been recently mobilized onto plasmids in bacteria.

### K62 appears to be unimportant for fungal virulence

Aerolysins are among the most potent bacterial virulence factors and are highly toxic to eukaryotic cells. As K62Ls are found in opportunistic fungal pathogens, we hypothesized that K62 could have a function beyond interference competition. *Drosophila melanogaster* larvae are attracted to food containing yeast and are sensitive to challenge by pathogenic fungi; hence, it was tested whether feeding larval flies with *S. paradoxus* expressing K62 could reduce the number of emerging adult flies (40, 41). Surprisingly, K62 expression decreased the number of emerging adult flies, but compared with the M62-o strain, the difference was not statistically significant (Fig. S15). *Galleria mellonella* larvae were also used to test the virulence of *S. paradoxus* Q62.5 with and without M62. After injection into the larvae, it was judged that neither strain could cause significant mortality compared with a buffer-only control (Fig. S15). Together, these data suggest that K62 is predominantly an antifungal toxin with little or no activity against higher eukaryotes, which contrasts sharply with the toxicity of bacterial aerolysins.

## DISCUSSION

This study identified the K62 killer toxin from *S. paradoxus* as the first member of a new family of aerolysin-like toxins in fungi. Only the hemolytic lectin from the fungus *Laetiporus sulphureus*, a member of the Basidiomycota, has been confirmed as a toxin that belongs to the aerolysin family and is not a sequence homolog of K62 (42). Previous bioinformatics studies have also identified sequence homologs of aerolysin family proteins across the tree of life, including fungi, but these have yet to be studied in detail (25). The identification of K62 and its homologs as a new group of toxins raises interesting questions about their contribution to fungal ecology and whether they have the potential to injure membranes of organisms beyond fungi. The overall conservation of the aerolysin pore-forming domain in all K62Ls would indicate that these proteins are likely pore-forming toxins.

Unlike other *Saccharomyces* killer toxins, K62 did not encode toxin immunity. Genome-encoded resistance indicated that the maintenance of M62 was independent of immunity factors that protect killer yeasts from their own toxins, as seen for K1, K2, and K28 (43–46). For example, the loss of the M1 satellite dsRNA (M1-o) leads to a loss of immunity to exogenous K1, whereas an M62-o strain remains resistant to K62. Such genome-encoded killer toxin resistance has been previously observed for the killer toxins zygocin and KP4 (47, 48). Although the mechanism of K62 resistance has not been determined, resistance to ionophoric killer toxins can be triggered by loss-of-function mutations in many genes, including those involved in cell wall biogenesis, which prevents toxin interaction with a primary cell wall receptor (20, 49, 50). As an aerolysin-like toxin, K62 immunity could be similar to bacterial aerolysins, which are blocked from attacking bacterial cells due to receptor specificity, membrane lipid composition, and extracellular activation by proteolytic processing.

Killer toxins and aerolysin toxins utilize proteolytic cleavage during toxin maturation to prevent collateral damage to the cell that produces the toxin. In the case of aerolysin and epsilon toxins, a C-terminal peptide is cleaved by proteases after extracellular export (51–53). Before cleavage, the C-terminal peptide aids in the proper folding of the aerolysin monomer (54). Once cleaved, aerolysin monomers can form oligomeric pre-pores. Similarly, *Saccharomyces* killer toxins require Golgi-specific proteases to complete maturation before being exported extracellularly as active killer toxins. Supporting the post-translational cleavage of K62, there was a decrease in the size of K62-HA monomers when expressed in yeast (~18.5 kDa) compared with bacteria (~28 kDa). The reduction in molecular weight is consistent with the cleavage of 9 kDa from the N-terminus of K62. The presence of a dibasic KR motif at K62 residue 111, on an exposed loop between the N-terminal and core aerolysin domain, suggests an endopeptidase cleavage of K62 by Kex2, similar to other *Saccharomyces* killer toxins. It is conceivable that the uncleaved K62 is an inactive protoxin activated in the Golgi before extracellular release. The dibasic motif at the boundary between the N- and C-terminal domains is also found in 57% of the K62 homologs. Such a post-translational modification would be unusual for an aerolysin-like toxin, as it would separate the aerolysin core domain from the N-terminal domain that aids receptor recognition. Cleavage and separation of the alpha and beta domains are common among killer toxins, such as K1 and K28, although the domains typically remain linked by disulfide bonds (55, 56). In contrast, the SMKT killer toxin is also cleaved, but domains remain associated through non-covalent interactions (57). There are also examples of aerolysin family proteins that lack significant N-terminal domains, such as monalysin (58). K62 homologs from bacteria also appear to have unusually short N-terminal domains, meaning that the aerolysin core domain of K62 and its homologs could be necessary and sufficient for pore formation.

The first step in aerolysin oligomerization occurs in solution with the formation of dimers, which is also the case for K62 (59). Once an aerolysin dimer reaches the target cell membrane, higher-order oligomers are assembled to enable the formation of the membrane-associated pre-pore structure (60). Oligomerization of the aerolysin family protein lysenin is predicted to occur via the consecutive addition of monomers to form “arcs” (61). Arcs are considered intermediates in the assembly of the circular pre-pore structure before pore formation. The observation of multiple K62 oligomers, when expressed by yeasts, could represent intermediates of arc/pre-pore assembly. In contrast, the high molecular weight complexes extracted from bacterial membranes could be K62 arcs, pre-pores, and pores. The formation of K62 pores is supported by the fact that K62 is highly toxic to *E. coli*. The stability of K62 oligomers is similar to that of aerolysin pore and pre-pore complexes, forming prion-like interactions between beta strands (60). However, treating high molecular weight K62 oligomers with urea represents the ordered disassembly of complexes, a reversal of arc/pre-pore oligomerization. Despite the sensitivity of a proportion of the oligomers to urea, it was not possible to completely disassemble all oligomers into monomers, which is consistent with the resistance of aerolysin pores to urea, suggesting a heterologous mixture of high molecular weight complexes (36). Unlike the complexes directly extracted from *E. coli* membranes, SDS-solubilized K62 oligomers purified by gel filtration were more easily dissociated into monomers by boiling with a reducing agent. This would suggest the purification of a distinct complex that is perhaps less stable than K62 pores. Whether high-molecular-weight K62 complexes over 250 kDa represent K62 pre-pores, pores, arcs, or a more complex mixture remains to be further investigated.

It is generally accepted that killer toxins are specific to fungal cells due to the requirement for fungal-specific cell surface receptors to bind target cells. However, studies have identified non-*Saccharomyces* killer yeasts capable of inhibiting bacterial growth (62–64). The ability of recombinant K62 expressed by *E. coli* to cause rapid cell death indicates that it may have a spectrum of activity that extends beyond fungi. The enrichment of K62 oligomers in bacterial membranes also supports the formation of aerolysin-like pores. The differences in the lipid composition of bacterial and fungal plasma membranes do not appear to prevent K62 intoxication, indicating that the toxin either binds and interacts with a subset of lipids that are common to both organisms (e.g., phosphatidylethanolamine) or has a broad specificity for membrane lipids during pore formation. Such a capacity to target different cell membranes would make K62 unique, as many aerolysin family proteins require specific surface receptors to bind their target cells prior to oligomerization and pore formation (65, 66). The high intracellular concentrations of K62 in the *E. coli* cytoplasm may have facilitated the assembly of pores without the need for specific receptors. Indeed, aerolysin-family proteins concentrate in membrane microdomains (e.g., lipid rafts) by binding to specific receptors, which enables efficient pore assembly, and could be analogous to the increased intracellular concentration of K62 during high-copy expression in *E. coli* (67). Further investigations would be necessary to assess whether K62 has the potential to injure bacterial cells when applied extracellularly and if it is capable of penetrating the outer lipopolysaccharides of the *E. coli* outer membrane.

Horizontal gene transfer plays a pivotal role in microbial evolution, particularly in diversifying and disseminating virulence factors, including toxins such as K62. Horizontal gene transfer has been documented in fungi at the level of individual genes and entire chromosomes, as seen in *Fusarium* species (68). Within fungi, the transfer of killer toxin genes between species has been linked to retrotransposition, plasmids, and large transposable elements named *Starships* (69–74). Moreover, the mobilization of killer toxins by M satellite dsRNAs and their associated totiviruses provides an additional mobilization mechanism, as the genomic integration of viruses has been observed in diverse yeast species (71). The frequent horizontal gene transfer of genomically encoded killer toxins related to K1, KHS, KP4, and zymocin is evinced by their sporadic and phylogenetically inconsistent distribution across fungal lineages (21, 73–75). The same inconsistencies are evident for K62 and its homologs, indicating that these proteins have also been subjected to horizontal gene transfer.

Horizontal gene transfer is a key driver in disseminating aerolysin family toxins, with the transfer from bacteria to eukaryotes having occurred on at least six occasions (76). The detection of K62L homologs on bacterial plasmids and dsRNA satellites indicates a plausible mechanism for their mobilization. The observed widespread and recurrent horizontal transfer of K62L genes supports their functional significance, suggesting that they likely confer a selective advantage. Indeed, many killer toxins are under positive evolutionary selection, indicative of allelopathy between species and an ongoing evolutionary arms race (21, 74, 77). Toxin genes are particularly well-suited to horizontal gene transfer because they often function as self-contained units, encoding a complete effector that, once expressed, can enhance competitiveness by inhibiting rival microorganisms. Together, these findings suggest that the true diversity of K62L genes may be far greater than currently appreciated. Broader, more comprehensive searches for these toxins will surely uncover more toxins, with widespread implications for fungal ecology and potentially human, animal, and plant health.

## MATERIALS AND METHODS

### Yeast and bacterial growth media

YPD media was used to maintain yeast cell lines and was composed of yeast extract (10 g L^−1^), peptone (20 g L^−1^), and dextrose (20 g L^−1^). The carbohydrate may be replaced by 20 g L^−1^ galactose or raffinose. Plates for killer assays were made by adding 2.99% wt/vol sodium citrate (Fisher Scientific #S279-3) and HCl (Fisher Scientific #A144C-212) to a pH of 4.6; 0.03% (wt/vol) methylene blue (Fisher Scientific # A18174) was added as a cell death indicator after autoclaving. Complete medium lacking uracil (CM -uracil) was used to select and maintain yeast cell lines that have plasmids. CM -uracil comprised 0.67% yeast nitrogen base without amino acids (Research Products International # Y20040-1000.0), 0.25% (wt/vol) amino acid mix minus uracil, and 2% (wt/vol) dextrose. LB medium was used to select and maintain *E. coli* cell lines (Fisher Scientific #BP1427-2). The appropriate antibiotic was added after autoclaving. Solid growth medium was made by adding 2% (wt/vol) agar (Sigma-Aldrich #A1296-1KG). All organisms used in this study are listed in Table S12.

### Killer assay

In total, 2 mL of MSY114 or another susceptible lawn strain was grown overnight in suitable culture conditions. This culture was then normalized to 6 OD_600_ units, and 5 µL was spread on the appropriate killer assay solid media in 1 mL sterile diH_2_O. The spread lawn was allowed to dry, and 5 µL spots of each killer yeast strain were plated on top of the lawn. The plates were imaged after 48–96 h of incubation at ~20°C.

### Double-stranded RNA extraction

Double-stranded RNAs were extracted from yeast strains following Okada et al. (78), with modifications stipulated in Crabtree et al. (79). Specifically, yeast cultures were grown in YPD and harvested by centrifugation at 3,000 × *g* for 5 min. A total of 450 µL of 2× STE (200 mM NaCl; 20 mM Tris-HCl, pH 8.0; 30 mM EDTA) and 0.5 µL β-mercaptoethanol were added to the cells and mixed before the addition of 50 µL of 10% (wt/vol) sodium dodecyl sulfate (SDS) and 350 µL phenol–chloroform–isoamyl alcohol (25:24:1) and vortexed for 3 min at 3,000 rpm; after centrifugation for 5 min at 20,000 × *g*, *~*500 µL of clarified soluble cell lysate was extracted, mixed with absolute ethanol (5:1), and transferred to cellulose columns. Column tubes were centrifuged at 10,000 × *g* for 30 s, followed by 350 µL of wash buffer (1× STE with 16% [vol/vol] EtOH). Columns were transferred to a new tube, 350 µL of 1× STE was added, and columns were centrifuged at 10,000 × *g* for 30 s. The eluted fraction was recovered, and 1 mL of absolute ethanol with 40 µL of 3 M aqueous sodium acetate (pH 5.2) was added, inverted, and then centrifuged at 20,000 × *g* for 15 min. The precipitated pellet is then air-dried and dissolved in 15 µL of water. The presence of dsRNAs was examined via agarose gel electrophoresis.

### Cycloheximide dsRNA satellite curing

Yeast strains to be cured of dsRNAs were grown overnight in 2 mL YPD broth, and 5 µL of the overnight culture was spotted onto YPD agar containing a final concentration of 4 µM cycloheximide. Cultures were incubated for 72 h at 30°C. Surviving cells were streaked to single colonies on fresh YPD, and dsRNA loss (curing) was confirmed by the loss of the killer phenotype and dsRNA extraction.

### Site-directed mutagenesis of plasmids

Mutagenic primers were designed using NEBbaseChanger using the wild-type sequence of M62 (https://nebasechanger.neb.com/). Primers were ordered lyophilized from MilliporeSigma and reconstituted with water to a final concentration of 10 µM for use in PCR. The PCR was set up according to the manufacturer’s instructions, with an initial denaturation (98°C for 3 min), followed by 25–30 cycles of denaturation (98°C for 30 s), annealing (primer-specific temperature for 30 s), and extension (72°C for 2 min). After cycling, there was a final extension step (72°C for 10 min). Successful PCR was verified with agarose gel electrophoresis to identify the product of the desired size. PCRs were subjected to DpnI restriction enzyme to remove the template DNA (New England Lab # R0176S) at 37°C. Phosphorylation was carried out by T4 Polynucleotide Kinase (New England Lab # M0201S), 10 mM ATP (New England Lab # P0756S), and 0.1 M DTT (Thermo Fisher # 707265 mL). A Qiagen PCR clean-up kit (Qiagen # 28506) was then used to purify nucleotides for a ligation reaction (New England Lab # M0202S). The entire reaction was used to transform *E. coli* cells (New England Lab # C3019H), following the manufacturer’s instructions. All primers used for site-directed mutagenesis are listed in Table S13.

### Gateway cloning

To create expression plasmids, 1 µL of the appropriate destination vector was combined with 1 µL of the entry vector and 0.5 µL of LR Clonase (Invitrogen, Gateway LR Clonase II Enzyme mix). The mixture was then incubated at room temperature for 7–16 h. After incubation, the mixture was used to transform 10-Beta *E. coli*. Single colonies were picked and used to inoculate 2 mL LB/ampicillin liquid medium. Inoculated cultures were grown overnight at 37°C for approximately 16 h. Liquid cultures underwent miniprep to purify the plasmid, and successful recombination was validated using restriction digest and agarose gel electrophoresis.

### Yeast transformation

In total, 2 mL culture of BY4741 or CRY1 was grown overnight in YPD at 30°C. This saturated culture was then used to inoculate a YPD culture to 0.2 OD_600_ units and grow to 1.0 to 1.5 OD_600_ units; 5 mL of culture was centrifuged at 3,000 × *g* for 2 min to pellet cells for each transformation. The liquid was decanted, and the cell pellet was washed by adding 5 mL of sterile deionized water (diH_2_O), vortexed to suspend the cell pellet, and then centrifuged under the same conditions again. The liquid was decanted, and the cell pellet was suspended in 100 mM LiAc, vortexed to suspend cells, incubated at 30°C for 10 min, and centrifuged under the same conditions to pellet the cells. The liquid was decanted, and the remaining cells were transferred to a 1.5 mL microcentrifuge tube and centrifuged at 8,000 × *g* for 1 min to pellet cells. All remaining liquid was removed with a micropipette. The following were overlaid: 240 µL 50% (wt/vol) PEG 3350 (polyethylene glycol), then 36 µL 1 M LiAc, 50 mg/mL ssDNA, and approximately 1 µg of vector DNA. The cells were then mixed with the overlaid components by pipetting and incubated at 30°C for 30 min to recover the cells. The transformation mixture was then heat shocked at 42°C for 20 min. The cells were pelleted at 8,000 × *g* for 1 min, and the supernatant was discarded. The cells were suspended in 200 µL sterile diH_2_O and plated on CM -uracil dextrose solid media. All plasmids used in this study are listed in Table S14.

### Genome integration of plasmids into *S. cerevisiae* CRY1

Yeast transformation with genome-integrating vector pAG306-GAL used the yeast transformation protocol; however, the plasmid had to be linearized before transformation. Vectors were prepared for genome integration by restriction digestion with a single cleavage in the *URA3* gene for homologous recombination. Linearized product was purified with a Qiagen PCR clean-up kit and eluted with 20 µL H_2_O. The entire volume was used as vector DNA in yeast transformation.

### *E. coli* transformation

In total, 25 µL of 10-Beta chemical competent *E. coli* (NEB 10-Beta Competent *E. coli*, High-Efficiency # C3019H) was combined with 1 pg to 100 ng of plasmid and subjected to the following treatment: 4°C incubation for 30 min, heat shock at 42°C for 30 s, 4°C for 5 min, 200 µL of outgrowth media added, and recovered at 37°C for 1 h. The mixture was then spread on LB/ampicillin plates (or the appropriate antibiotic) and incubated at 37°C overnight.

### Sample preparation for western blotting (yeast)

Each strain was cultured overnight in 2 mL of CM -uracil dextrose. The saturated culture was used to inoculate 5 mL of CM -uracil raffinose to 0.02 ODs of each strain and allowed to grow for 16 h. After this incubation period, the strains were inoculated with 1 mL of 20% galactose or dextrose and grown for an additional 4 h, with the option to extend up to 8 h to ensure cells reach at least 1.5 ODs. Cells were pelleted at 1,000 × *g* for 5 min, suspended in sterile water, pelleted again to wash, and then normalized to 1.5 ODs. Cells could be flash-frozen in liquid nitrogen and stored at −20°C.

Cell lysis was carried out by adding 100 µL of cracking buffer (8 M urea, 5% wt/vol SDS, 40 mM Tris-HCl [pH 6.8], 0.1 mM EDTA, 0.4 mg/mL bromophenol blue, 120 mM βME, 10 vol/vol Halt Protease and Phosphatase Inhibitor Cocktails (Thermo Scientific # 78440) and 70 µL of glass beads 500–750 µm (Acros Organics #397641000) per sample, followed by incubation at 70°C for 10 min. The mixture was then vortexed at 3,000 rpm for 1 min (disruptor genie, scientific industries), centrifuged at 18,000 × *g* for 5 min, and 45 µL of the supernatant was transferred to a new 1.5 microcentrifuge tube and set aside. Each sample was incubated at 98°C for 3 min, followed by vortexing at 3,000 rpm for 1 min, centrifugation at 18,000 × *g* for 5 min, and transferring 45 µL to the previously set-aside sample. Finally, the samples could be flash-frozen in liquid nitrogen, stored at −20°C, or used immediately in a western blot.

### Sample preparation for western blotting (*E. coli*)

BL21 pLysS (DE3) *E. coli* containing pDEST14 K62-HA expression plasmids were grown in LB media with chloramphenicol and ampicillin to an OD of 0.9-1.1 and induced with 100 nM IPTG for 1 h; 1 OD worth of cells was collected, pelleted, and frozen at −20°C. Cells were suspended in SDS running buffer and Laemmli buffer, boiled, and loaded onto SDS-PAGE gels for western blot analysis.

### *E. coli* membrane fraction preparation

A 500 mL culture of *E. coli* was induced for 1 h with 100 µM ITPG, and the membrane fraction was isolated. Induced cells were suspended in 10–13 mL PBS, pH 7.4, with 1 mM PMSF and one cOmplete mini protease inhibitor tablet (Pierce). After suspension, an additional 1 mM PMSF was added with 40 units DNase I. Cells were lysed by freeze/thaw (three cycles between liquid nitrogen and 30°C until suspension thawed). This material was then passed through an 18-gauge needle to shear DNA. This treated material was centrifuged for 12 min at 21,000 rpm at 4°C in a S100AT5 rotor to remove large debris. The supernatant from this step was centrifuged for 45 min at 53,000 rpm at 4°C in the S100AT5 rotor. The supernatant from this centrifugation step was kept and considered a cytoplasmic fraction. The pelleted material was suspended in a total volume of 6 mL PBS, pH 7.4, and centrifuged for an additional 45 min at 53,000 rpm at 4°C in a S100AT5 rotor. The supernatant was removed, and the remaining pellet was suspended in 2 mL PBS, pH 7.4. This final material was considered the membrane fraction.

### K62 gel filtration

Gel filtration chromatography was performed on an NGC chromatography system (BioRad) using a HiLoad 16/600 Superdex 200 pg column. The chromatography was performed using a 50 mM sodium phosphate, 150 mM sodium chloride buffer, pH 7.2. One milliliter of dialyzed, treated membrane fraction was loaded per chromatography run. Sizes were determined using gel filtration standards (BioRad # 1511901) with an additional 0.5 mL 2 mg mL^−1^ Dextran Blue added to the column run.

### Western blotting

Samples were separated by SDS-PAGE and transferred to nitrocellulose membranes by Trans-Blot Turbo Transfer System using the preprogrammed mixed molecular weight transfer protocol (1.0 A, 25V, 7 min) or, for membrane preparations, 1.3 A, 25 V, 45 min. Ponceau stain was used to verify effective transfer, and the sample was imaged using an Amersham Imager 600. Membranes were blocked with 3% non-fat milk in TBST (1 × TBS, 0.1% [vol/vol] Tween-20) for 30 min. Rat anti-HA-HRP (3F10, Sigma-Aldrich # 12013819001; 1 in 2,000 TBS or 1 in 4,000 for probing membrane preparations) and mouse anti-GAPDH-HRP (GA1R, Thermo Scientific MA5-15738-HRP; 1 in 1,000 TBS) were used to probe for HA epitope and GAPDH loading control, respectively, for 30 min. The membrane was washed with gentle rocking in TBST for 1, 1, 2, and 3 min.

### Ammonium sulfate precipitation of the K62 killer toxin

Killer yeast strains were cultured overnight in 2 mL of YPD, pH 4.6, at 25°C. The overnight culture was used to inoculate a 10 mL culture, which was then grown overnight and used to inoculate a 25 mL culture the next day, which was grown overnight; 25 mL cultures were then removed from shaking and left at room temperature for a day. Each culture was then sterile filtered through 0.45 µm PES into 5 mL tubes and combined 1 to 1 with supersaturated ammonium sulfate. The solution was left overnight at 4°C. The next day, each solution was centrifuged at 10,000 × *g* for 20 min. The supernatant was decanted, tubes were spun down, and the remaining liquid was removed via pipetting; 10 µL of YPD, pH 4.6, per 5 mL tube was used to suspend the protein and used in other assays.

### *Galleria mellonella* pathogenicity assay

*G. mellonella* larvae were maintained under standardized conditions to ensure experimental reproducibility and were ordered and shipped on ice from https://www.premiumcrickets.com/. Upon arrival in the laboratory, larvae were stored in the dark at 17°C in wood shavings for at least 48  h. Before injection, larvae were screened for uniformity in mass (175–225  mg), length, and responsiveness to tactile stimulation. Individuals displaying advanced melanization or signs of morbidity were excluded. Selected larvae were acclimated at 25°C for 16 h before manipulation. Larvae were immobilized using a restraining apparatus made from 250 µL pipette tips, with the posterior end inserted into the wider half and the narrower end used to gently secure the animal, leaving the last left proleg exposed (80). Injections were performed using a Hamilton 700 µL syringe, disinfected before each injection series by three rinses with 70% ethanol, followed by sterile deionized water. The needle was inserted at a shallow angle (10°–20°) beneath the cuticle to avoid the midgut, and the total volume of inoculant was delivered into the hemocoel (<5 mm depth). Control groups were injected with sterile 1× PBS. The syringe was cleaned every 10 injections and between different inoculant conditions by ethanol wiping and sterile water rinsing. Post-injection, larvae were placed in 9 cm petri dishes lined with sterile paper towels (maximum 10 larvae per dish), sealed with Parafilm, and incubated in the dark at 25°C. Mortality was assessed daily over 5 days based on movement and tactile response. After the assay, larvae were euthanized at −20°C for 24 h and disposed of by autoclaving.

### *D. melanogaster* pathogenicity assay

Fly food vials (1 L) were prepared 1 day before experimentation, initially without yeast supplementation. Fly food medium (Bloomington formulation, Genesee) in each vial was liquefied via microwave heating. Melted food was maintained at 50°C in a water bath. Different yeast treatments were subsequently added to sets of 10 vials each. Drosophila embryos were collected and suspended in phosphate-buffered saline (PBS) and transferred to the resolidified food vials containing yeast cells in a randomized order. Vial positions were randomized within incubation trays before placement in a 25°C incubator. Following a 2-week incubation period, total adult flies were recorded for each vial.

### *D. melanogaster* toxicity statistical methods

All analyses were done using Program R (2023). The distribution of progeny numbers under each condition was evaluated for normality with a Shapiro-Wilk test, and homogeneity of variance was assessed with an HOV test. The data were not normally distributed, or the variances were unequal. Therefore, a Kruskal-Wallis test was performed, followed by pairwise Wilcoxon rank-sum tests to identify statistically significant differences (*P* < 0.05) in progeny counts between toxic and non-toxic conditions.

### Phylogenetic analysis

A search for similar sequences in NCBI (https://www.ncbi.nlm.nih.gov/) was carried out with PSI-Blastp, with the results filtered by accession length (75% [204 amino acids] to 150% [408 amino acids] of K62). The protein sequences were aligned with Clustal Omega. Amino acid substitution models were generated according to the Bayesian information criterion (BIC), with VT + R4 being the chosen model. Maximum likelihood (ML) phylogenetic trees were reconstructed in IQTREE2, with 10,000 ultrafast bootstrap replicates (tree completed after 111 iterations, LogL: −38,038.315) (81). The final trees were edited in FigTree v.1.4.3 and iTOL (82). The phylogenetic tree file in Newick format is provided in File S1.

### Structure prediction

All predicted structures presented in this study were generated using AlphaFold2 (version 2.2.0) with default parameters. Structure prediction was performed via the singularity.py script on the University of Idaho’s Research Computing and Data Services platform. For monomer predictions, five models were generated for each sequence with amber relaxation, and pLDDT was used to select the top-ranked model for further analysis. The same protocol was used for each multimer prediction, except that 25 models were generated instead of 5.

### Molecular dynamics simulation

MD simulation was carried out using GROMACS version 2018.3 using a protocol adapted from the GROMACS tutorial, which was also the source for MDP files (83). Protein was solvated in a dodecahedron box using a 10 Angstrom layer of TIP3P water, and ions were added to neutralize the protein’s charge. The AMBER99SB-ILDN force field was used to parameterize protein residues and ions. Energy minimization was carried out using steepest descent for 50,000 steps or until the maximum force was less than 1,000 kJ/mol/nm. NVT and NPT were performed each for 50,000 steps using 2 fs integrals, bringing the protein to 300 K and 1 bar. The production simulation was run for 100 ns and analyzed using the GROMACS package tools. During simulation, the pressure was maintained using Parrinello-Rahman coupling, the LINCS algorithm was used to constrain bonds to ideal lengths, V-rescale was used to control temperature, and electrostatic and van der Waals cutoffs were 1.2 nm and 1.0 nm, respectively.

### FoldX

FoldX version 5.0 was used to calculate the ΔΔG of stability of cysteine point mutations to alanine on the amber relaxed top-ranked model (84). Before mutation and ΔΔG calculation, the model was subjected to FoldX RepairPDB six times, which has been shown to improve the accuracy of ΔΔG calculation (85). The PositionScan command was used to create mutant structures and calculate energy.

## SUPPLEMENTAL MATERIAL

File S1 mbio.01425-25-s0001.txt Phylogenetic relationship of the K62 family of proteins in Newick format.

Supplemental figures mbio.01425-25-s0002.pdf Fig. S1-S15.

Legend mbio.01425-25-s0003.docx File S1 legend.

Supplementary tables mbio.01425-25-s0004.pdf Tables S1-S14.

ASM does not own the copyrights to Supplemental Material that may be linked to, or accessed through, an article. The authors have granted ASM a non-exclusive, world-wide license to publish the Supplemental Material files. Please contact the corresponding author directly for reuse.
