# Supplementary material for: The Saccharomyces killer toxin K62 is a protein of the aerolysin family
Source: mBio. 2025 Nov 11;16(12):e01425-25. doi: 10.1128/mbio.01425-25 (PMC12691639; doi:10.1128/mbio.01425-25)
Supplement: Supplemental figures — Fig. S1-S15. [file mbio.01425-25-s0002.pdf]

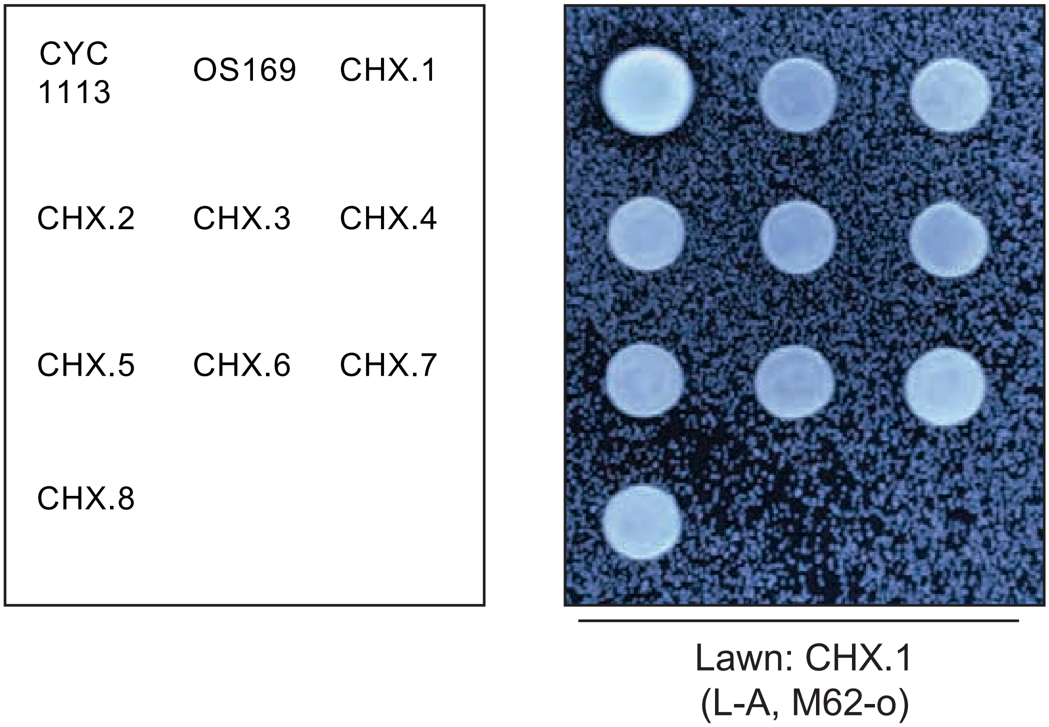

Figure S1

Curing of Q62.5 does not trigger K62 susceptibility. Killer phenotype assay using a cycloheximide (CHX) cured strain of OS169 lacking M62 (M62-o). A K2 killer yeast (CYC1113), K62 killer yeast (OS169), and M62-o strains (CHX.1 to CHX.8) were spotted at high density. Only CYC1113 was capable of weakly inhibiting the growth of the M-o lawn strain.

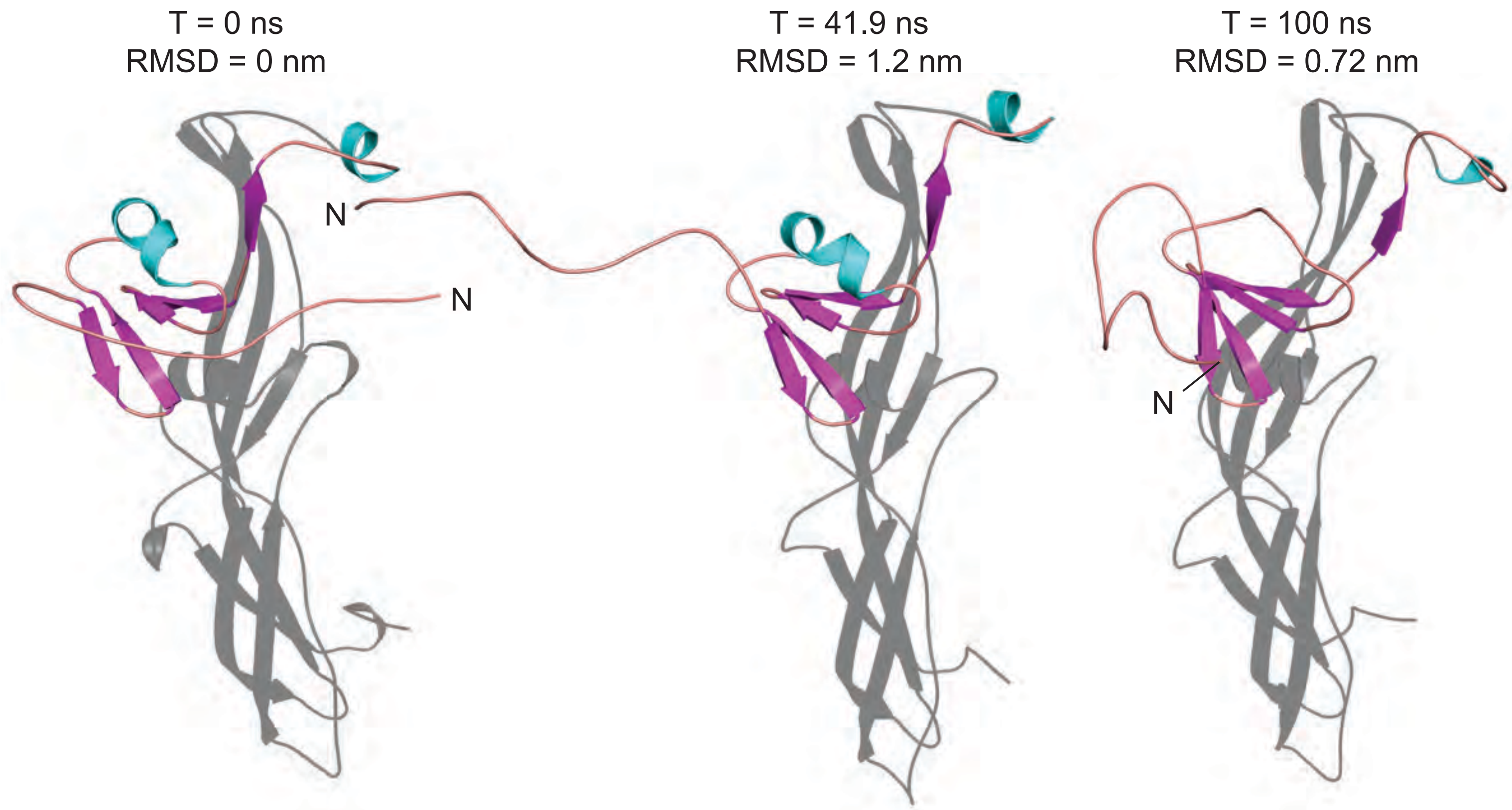

Figure S2

The N-terminus is highly mobile during MD simulation. The N-terminal domain is colored by secondary structure: sheets in purple, helices in cyan, and loops in orange. The aerolysin core domain is grey, which allows for a focus on the N-terminal domain.

A

K62	TWSYDVSIGISWEVISASVDYSISQS
Parasporin-2	TGFKFTSKLSIKKVFEIGGEVSFSTTIG
Aerolysin	KVTTKNKFKWPLVGETELSIEIAANQS
Epsilon	SIQATAKFTVPFNETGVSLTTSYSFANT

B

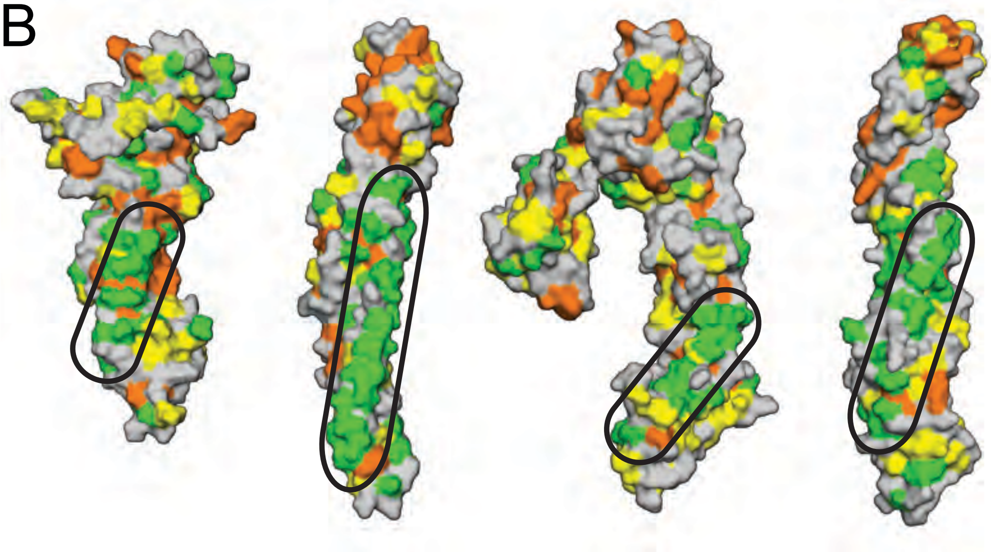

Figure S3

Sequences of known aerolysin insertion loops and K62. (A) Residues are colored green for polar, blue for positive, red for negative, and black for hydrophobic. (B) Surface properties sheets 2 and 3 of the K62 model, parasporin-2, and structural neighbors. Left to right: K62, parasporin-2 (2ztb), aerolysin (1pre A), and epsilon (1uyj A). Surface representations colored by residue: Serine and threonine in green, aromatics (phenylalanine, tyrosine, and tryptophan) in orange, hydrophobic residues (alanine, valine, proline, isoleucine, leucine, cysteine, and methionine) in yellow.

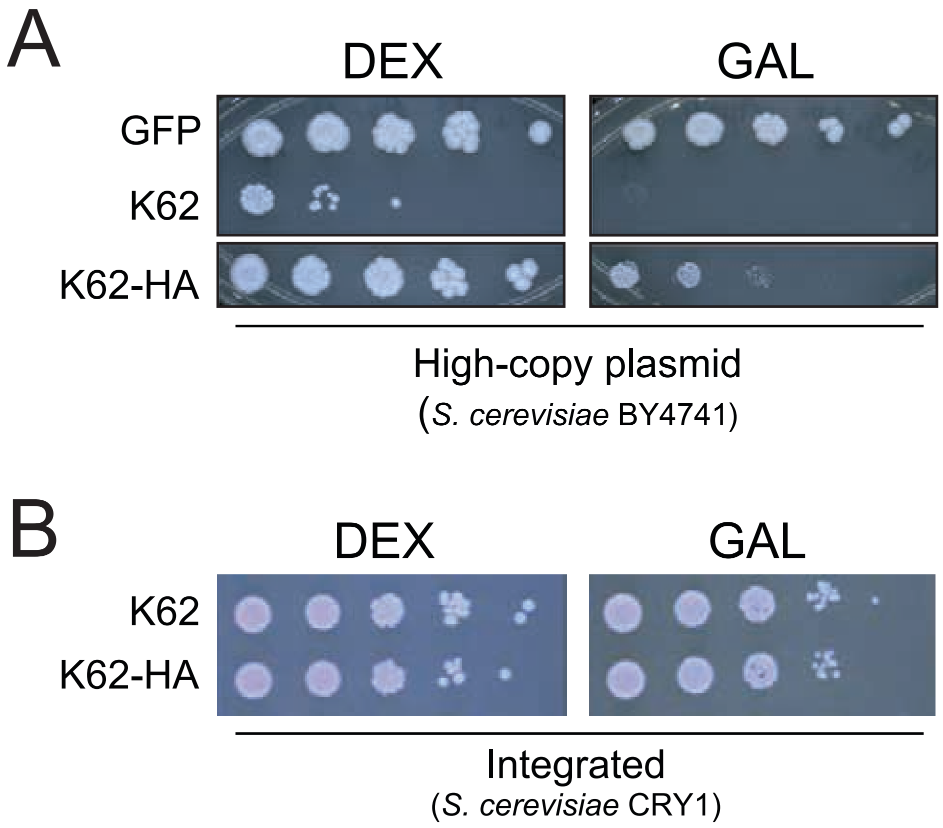

Figure S4

Toxicity observed after overexpression of K62 is strain-specific. (A) Galactose-inducible expression of K62 with or without a C-terminal HA-tag by *S. cerevisiae* BY4741 from a high copy episomal plasmid. (B) Galactose-inducible expression of K62 with or without a C-terminal HA-tag by *S. cerevisiae* CRY1 from a genome-integrated plasmid.

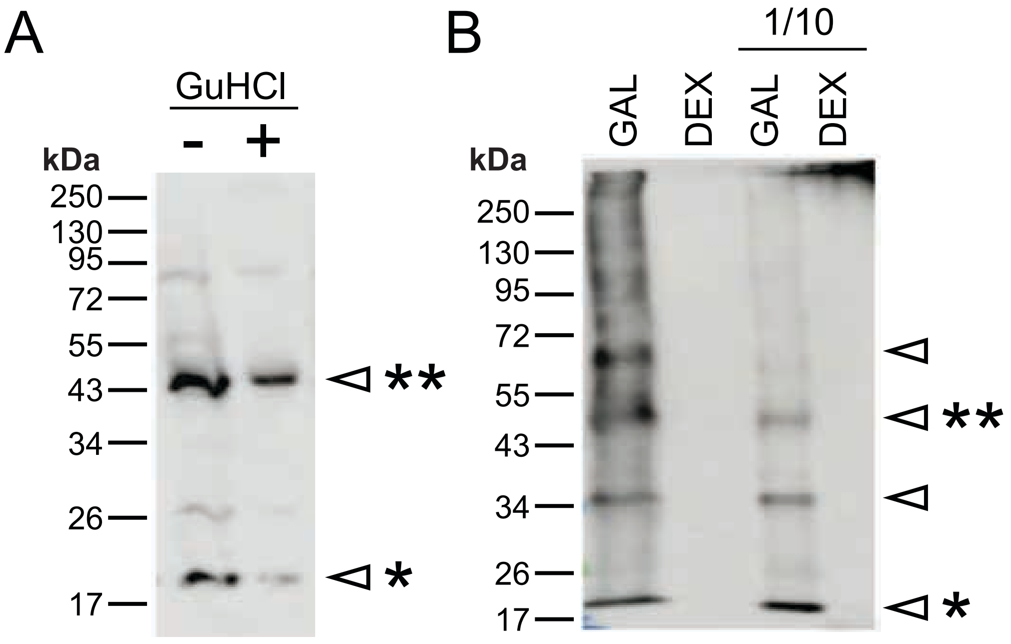

Figure S5

K62-HA forms a dimer in solution and is detectable in cell-free supernatant. (A) Guanidine hydrochloride was used in an attempt to denature dimers of K62-HA. (B) Growth media containing galactose or dextrose (D) were used to grow cells with a plasmid encoding K62-HA. The culture medium was filtered to remove cells and proteins precipitated with trichloroacetic acid. SDS-PAG and western blot analyzed undiluted and one-tenth dilutions of induced (GAL) and uninduced (DEX) precipitate.

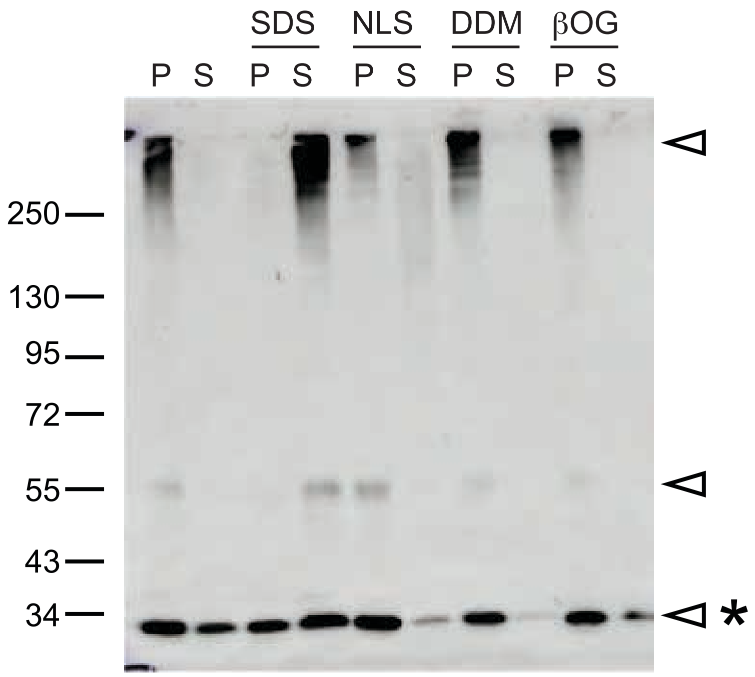

Figure S6

Membrane-associated high molecular weight oligomers of K62-HA can be solubilized by detergent treatment. Membrane fractions with oligomers of K62-HA treated with different detergents (SDS, sodium dodecyl sulfate NLS, N-lauryl sarcosine DDM, dodecyl maltoside beta-OG, Octyl beta-d-glucopyranoside). Pellet and soluble supernatant fractions were assayed for K62-HA by western blotting.

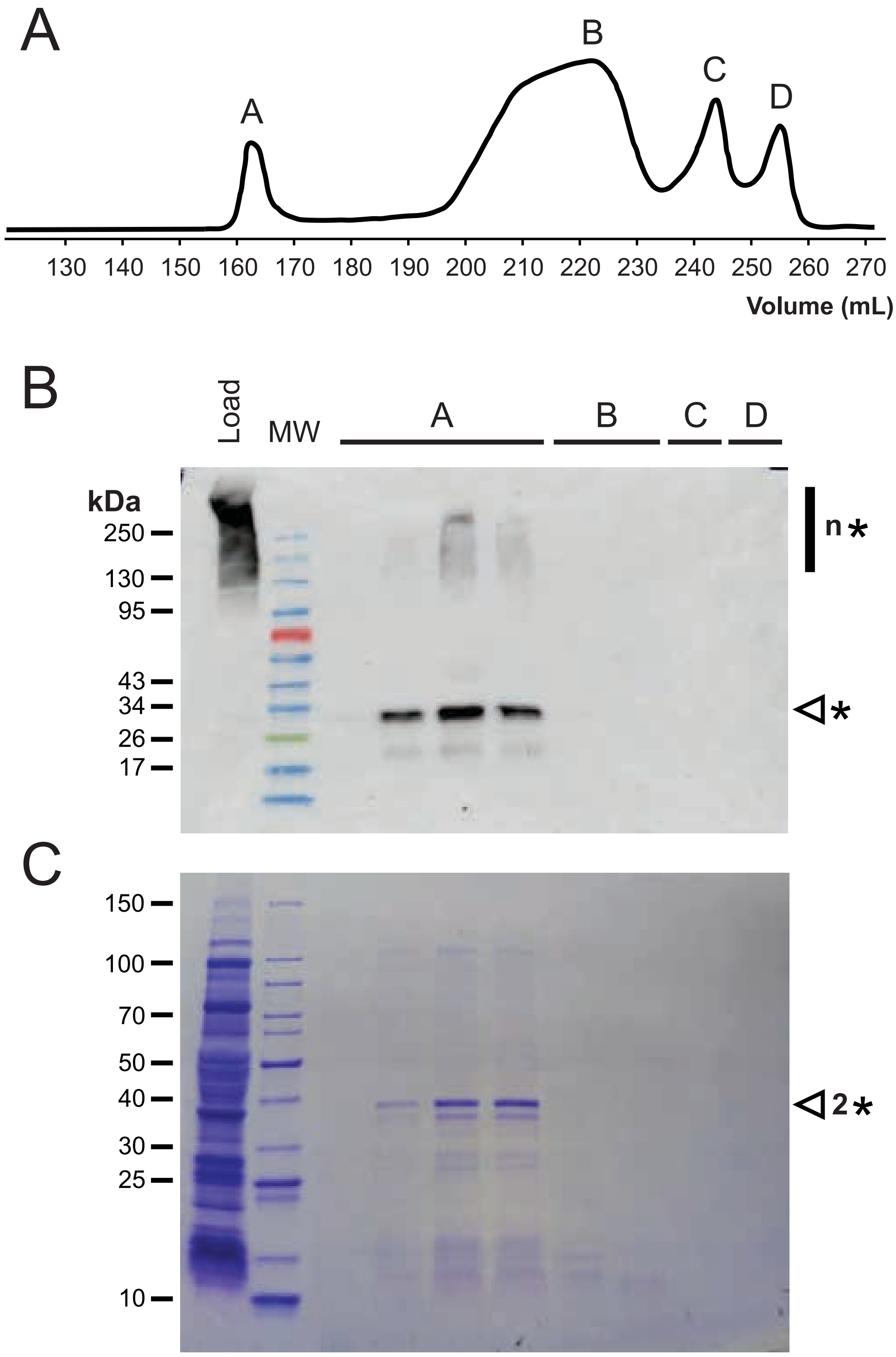

Figure S7

Purification of High Molecular K62-HA Complexes by Gel Filtration. (A) Size exclusion column elution profile of solubilized K62-HA complexes extracted from *E. coli* membranes (ultraviolet trace (280 nm)). Numerals indicate the major elution peaks analyzed by denaturing SDS-PAGE, followed by (B) western blotting. The solubilized K62-HA complexes (input) were probed with anti-HA primary antibody, revealing signals consistent with the majority of K62-HA as monomers of ~35-40 kDa (*) with oligomers >250 kDa (n*).

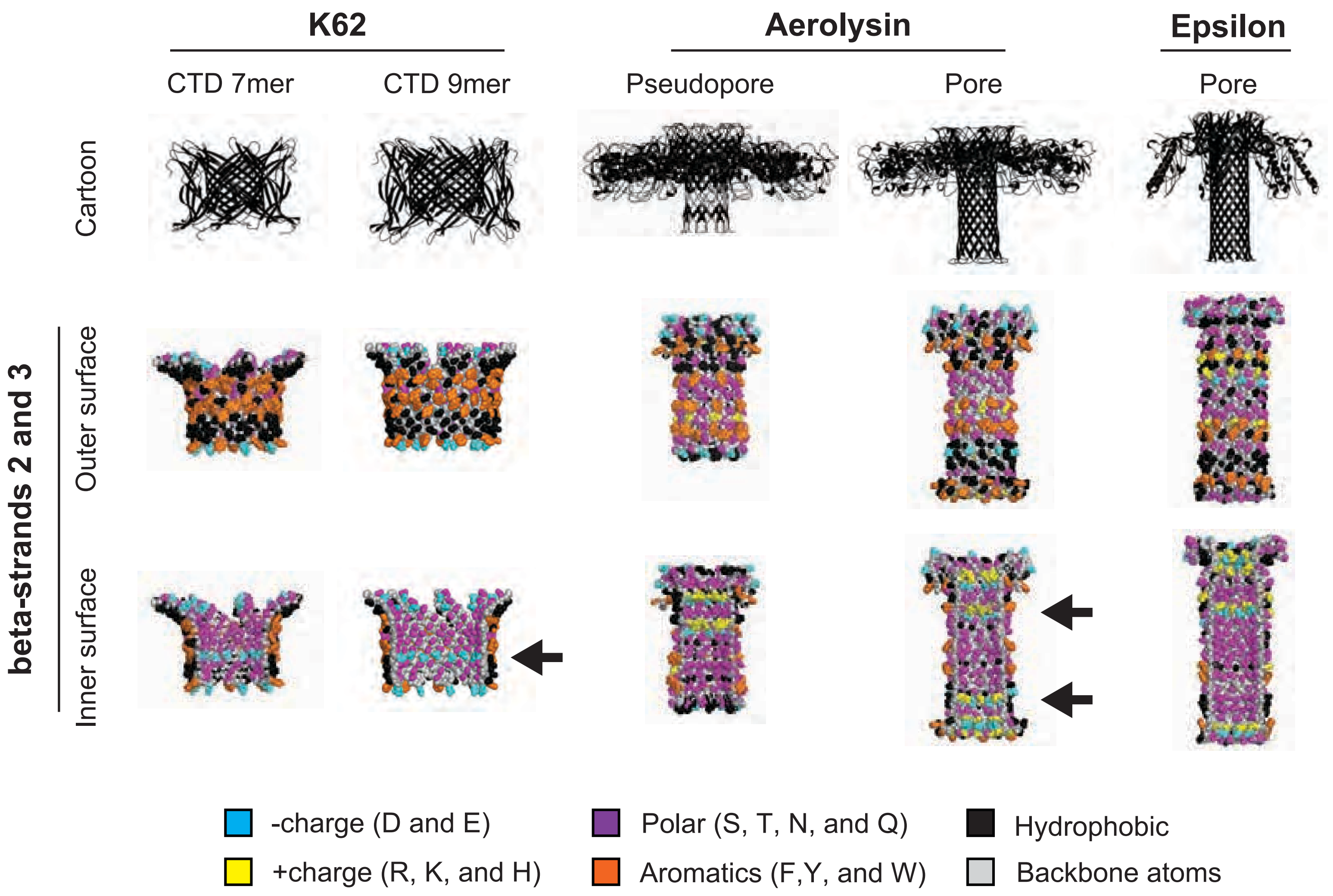

Figure S8

AlphaFold2 predictions of K62 C-terminal domain oligomerization. K62 C-terminal domain AlphaFold2 predictions compared to resolved structures of aerolysin quasi-pore (pdb: 5jzw), aerolysin pore (pdb: 5jzt), and epsilon pore (pdb: 6RB9). Top: Cartoon representation comparing the overall structure. Middle: Representations of the exterior surface of beta strands 2 and 3 form the beta-barrel within each structure. Spheres are colored based on the properties of the residues. Bottom: Cross-section depicting the interior of each structure s beta barrel using the same representation as the middle panel. Arrows indicate the ring of aspartic acid residues D171 and D186 in the K62 pore model and R220 and K238 residues.

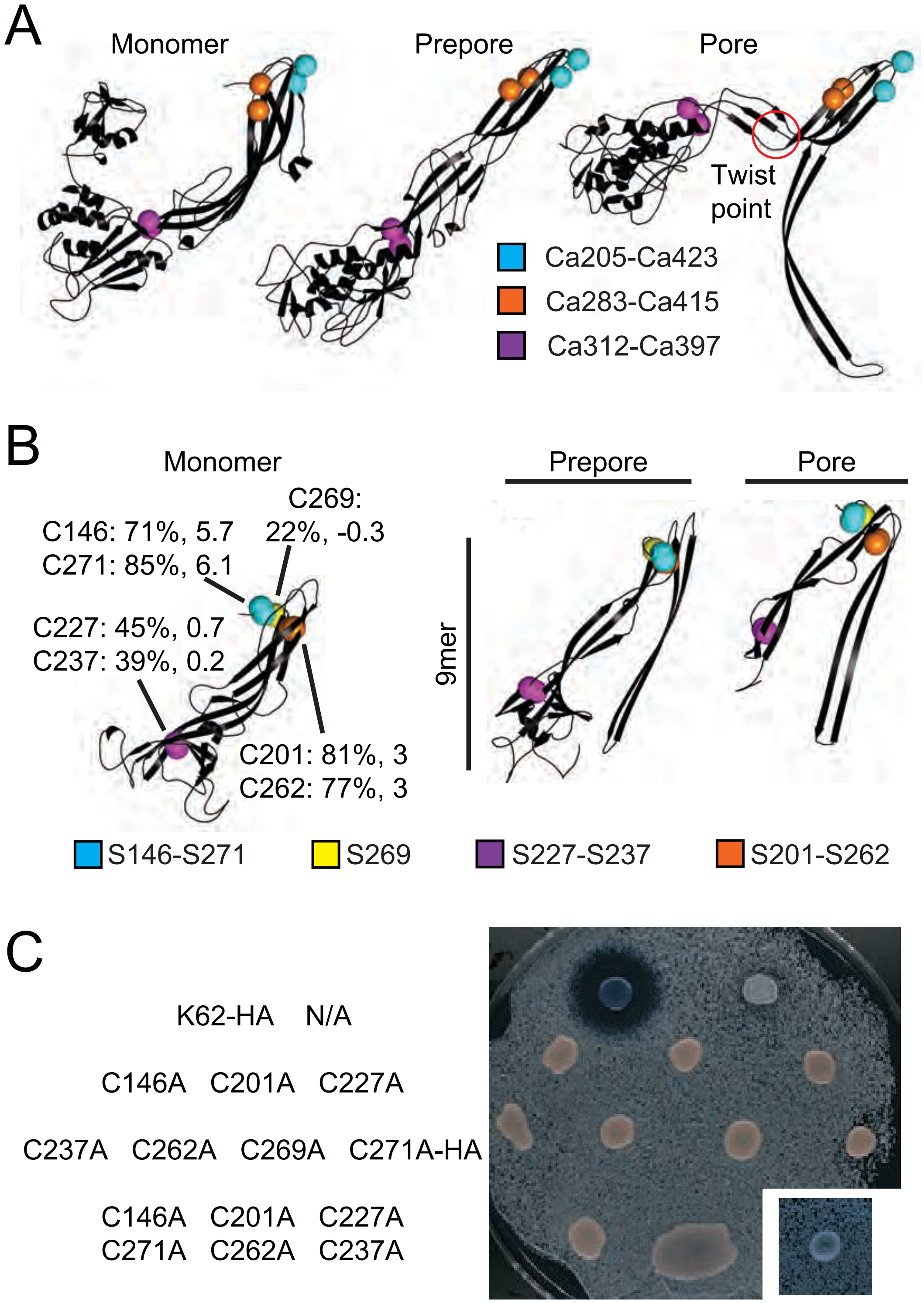

Figure S9

Predicted disulfide bonds in K62 are unlikely to constrain conformational changes during pore formation but are essential for K62 antifungal activity. (A) Aerolysin crystal structures of monomer (1pre), pre-pore subunit (5jzhA), and pore subunit (5jztA) with the relative position of three K62 cysteine pairs highlighted as spheres (Ca = alpha carbon). (B) Alphafold2 predictions of K62 with cysteine sulfur atoms predicted to form disulfide bonds are highlighted as spheres. For each K62 cysteine, the conservation across 1,000 homologs is expressed as a percentage. (C) Killer plate assay showing the loss of K62 antifungal activity after the mutation of single cysteines or cysteine pairs relative to wild-type K62.

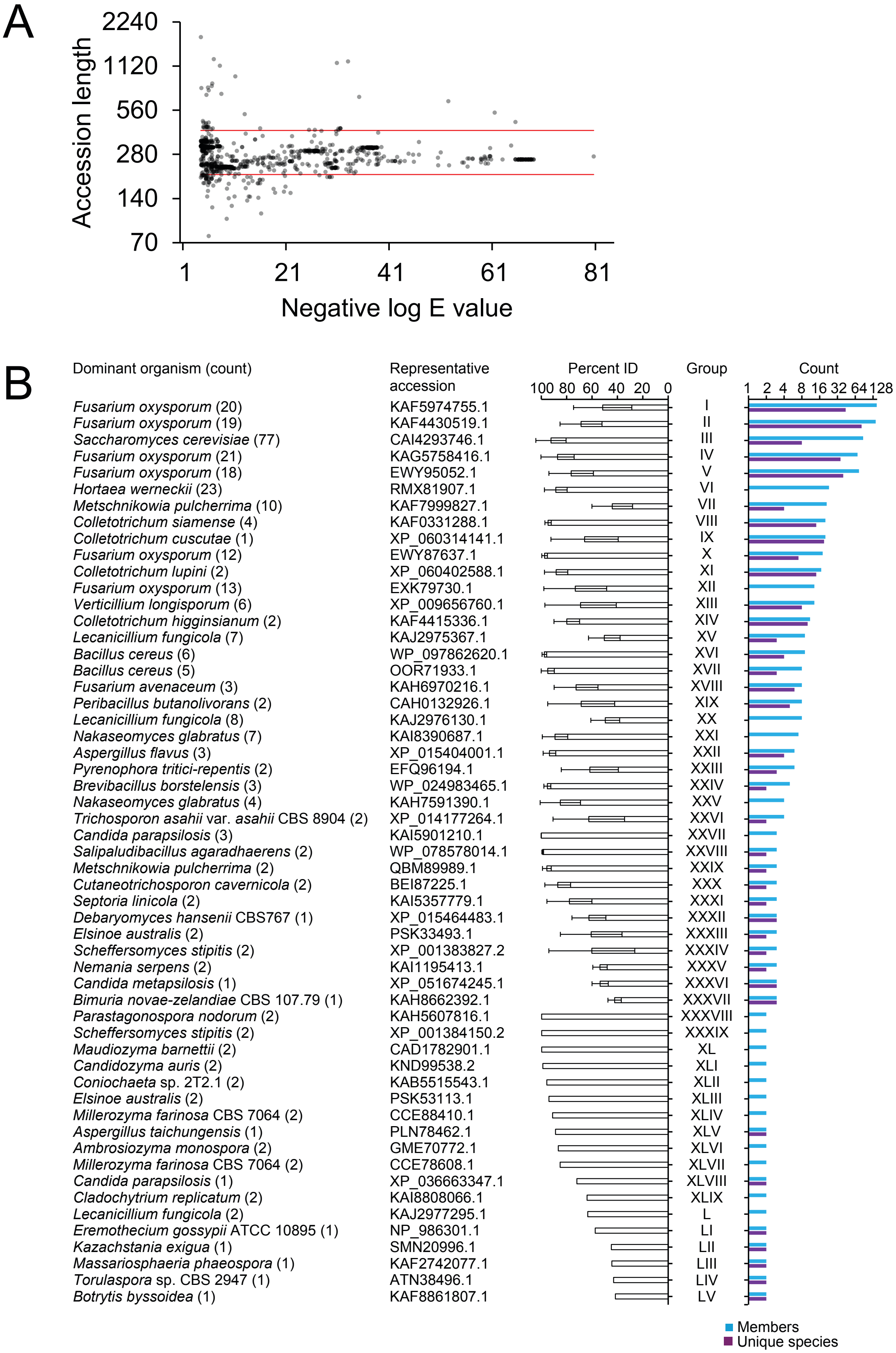

Figure S10

Bioinformatics summary of PSI-BLAST hits and 40% grouping analysis. (A) A plot of PSI-BLAST hits. Red lines mark upper and lower accession length boundaries for sequences used in downstream analysis. (B) Grouping by percentage identity was used to determine representative accessions for making structural predictions.

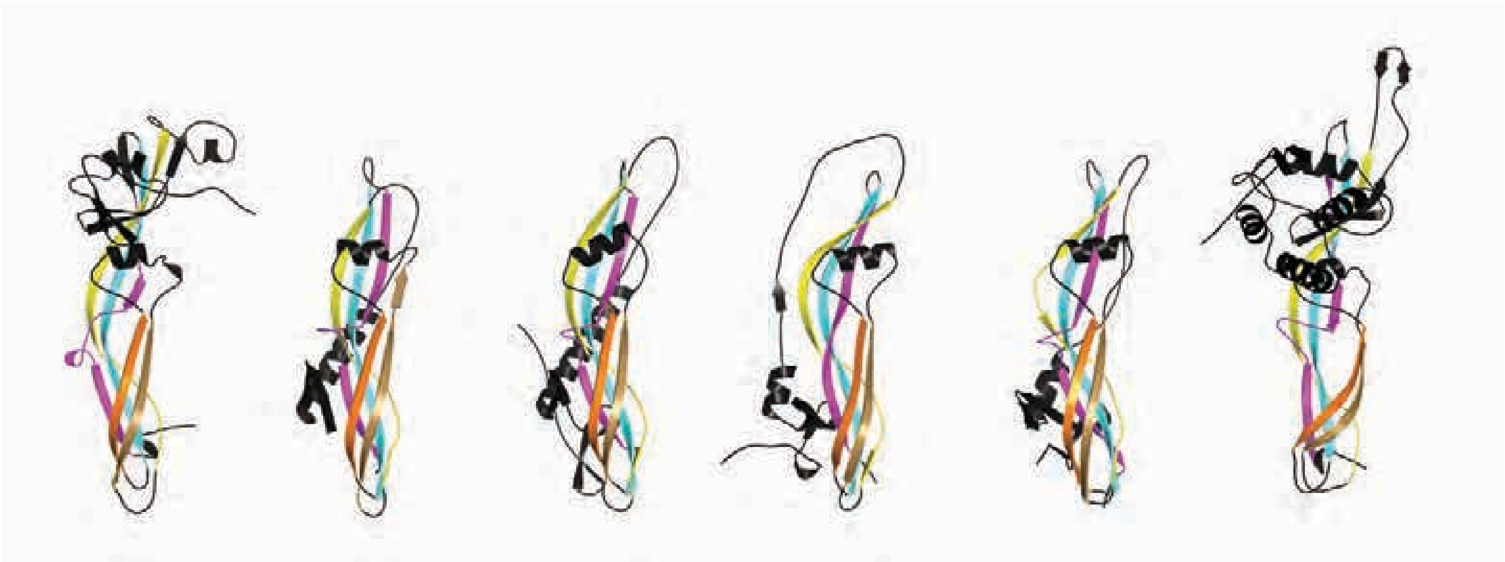

Figure S11

Representative bacterial AlphFoldF2 tertiary structure predictions. C-terminal beta sheets are colored according to beta sheets of the aerolysin motif, B1: Yellow, B2: Sand, B3: Orange, B4: Cyan, and B5: Magenta. The N-terminal domains are colored black. From left to right, the structures are K62, *Bacillus cereus* group 1 (OOR71933.1), *Bacillus cereus* group 2 (WP_097862620.1), *Brevibacillus* group (WP_024983465.1), *Salipaludibacillus* group (WP_078578014.1), and *Staphylococcus equorum* (MDN6570107.1). For clarity, the first 25 amino acids of all structures were omitted.

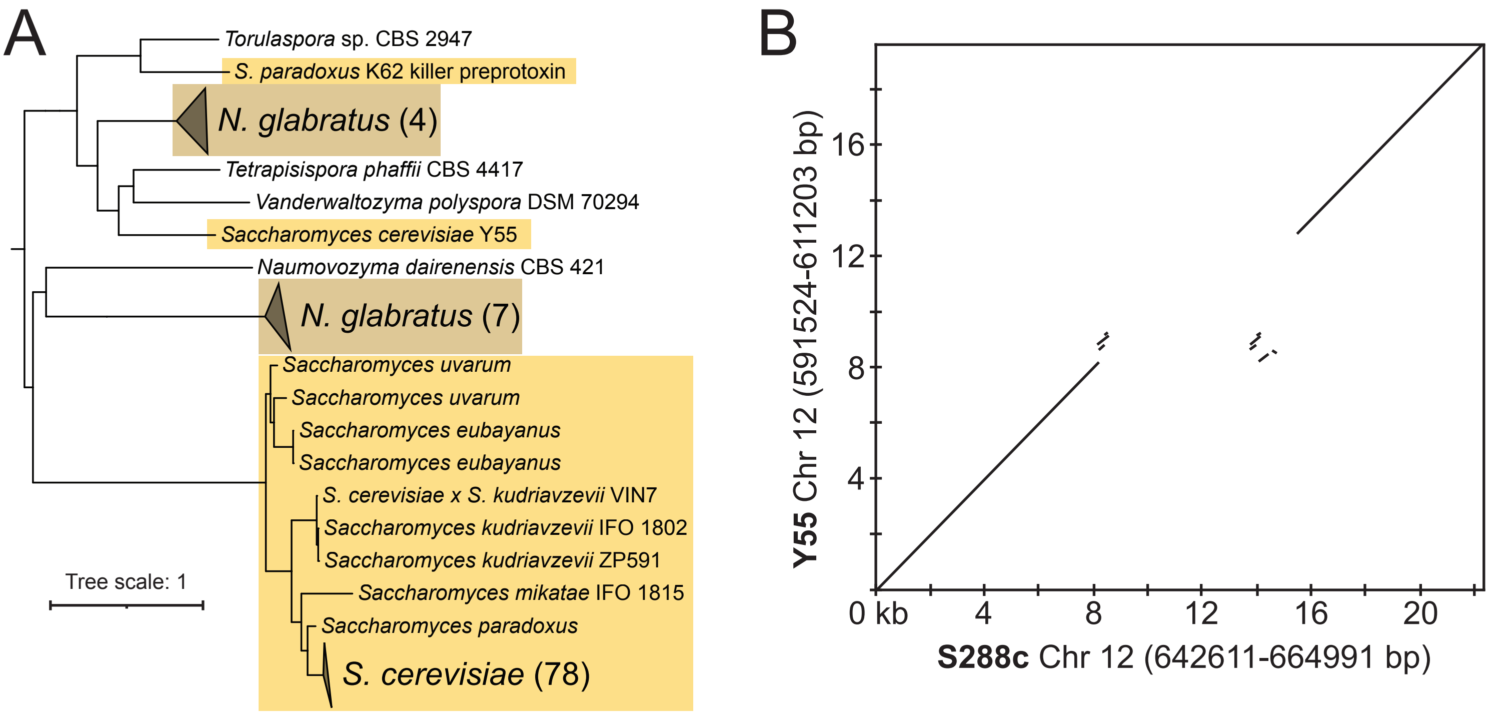

Figure S12

Vertical and horizontal gene transfer of K62 homologs in the *Saccharomyces* genus. (A) Local phylogeny of *KTA1* and *KTA2* in the yeasts. Colors correspond to Figure 6, with yellow for *Saccharomyces* and brown for *Nakaseomyces*. (B) Pairwise alignment of the region from Chromosome 12 of Y55 and S288c showing the insertion of *KTA2*.

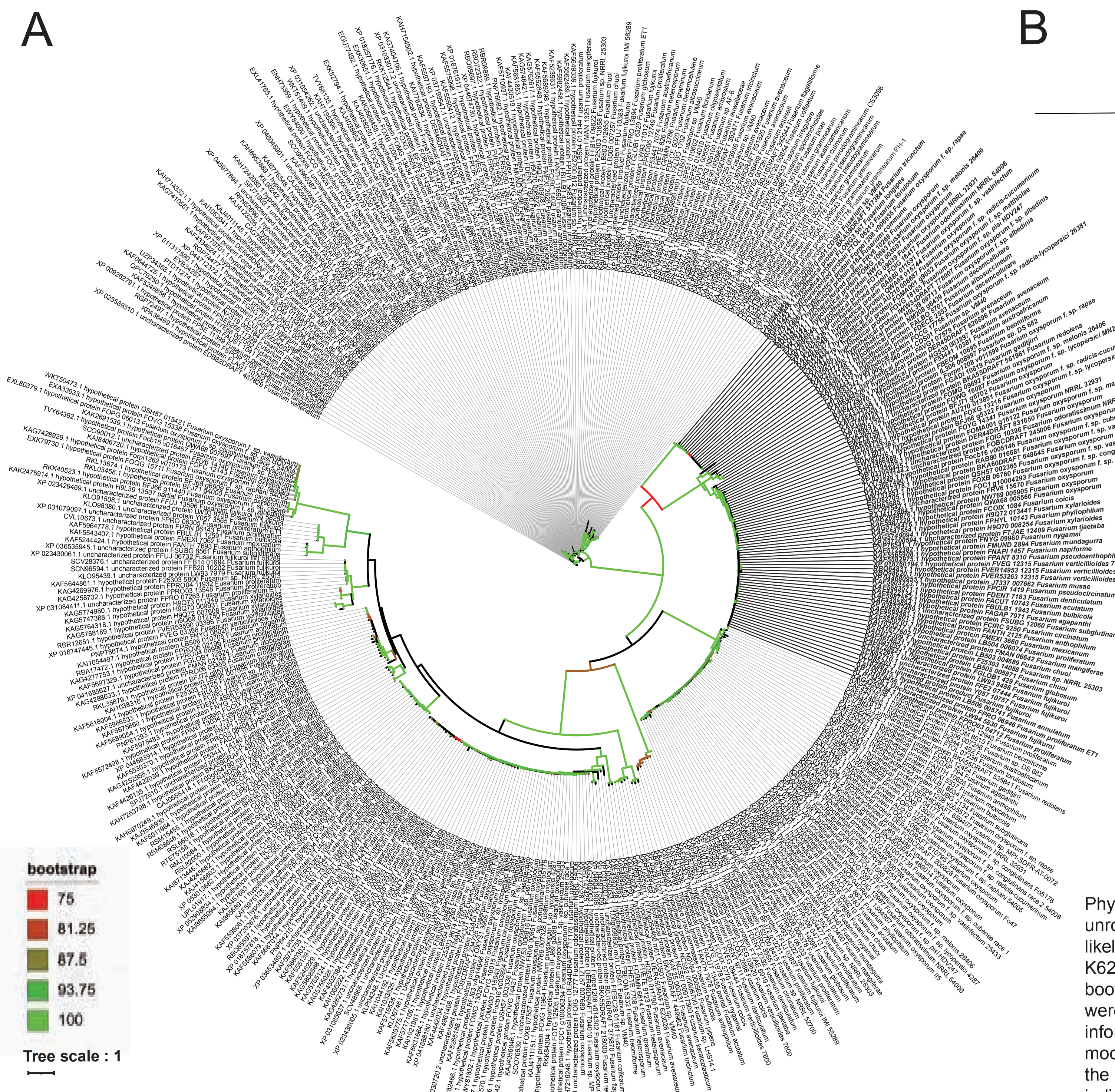

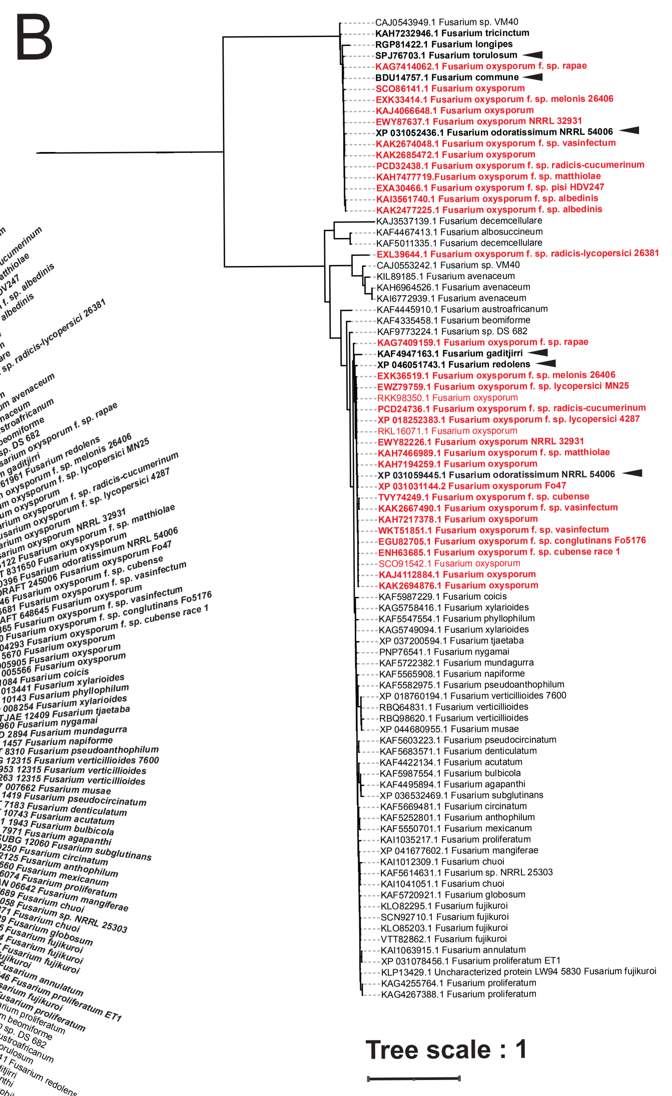

Figure S13

Phylogenetic tree of K62Ls from *Fusarium*. (A) An unrooted phylogenetic tree showing a maximum likelihood model based on amino acid sequences of K62 homologs from *Fusarium*, calculated with 10,000 bootstrap iterations. Amino acid substitution models were determined according to the Bayesian information criterion to determine that the VT+R4 model fit the assembled data. Bold italic text indicates the branches extracted for panel (B). Arrowheads indicate K62L proteins that show phylogenetic discordance and are nested within sequences derived from *F. oxysporum* (highlighted in red).

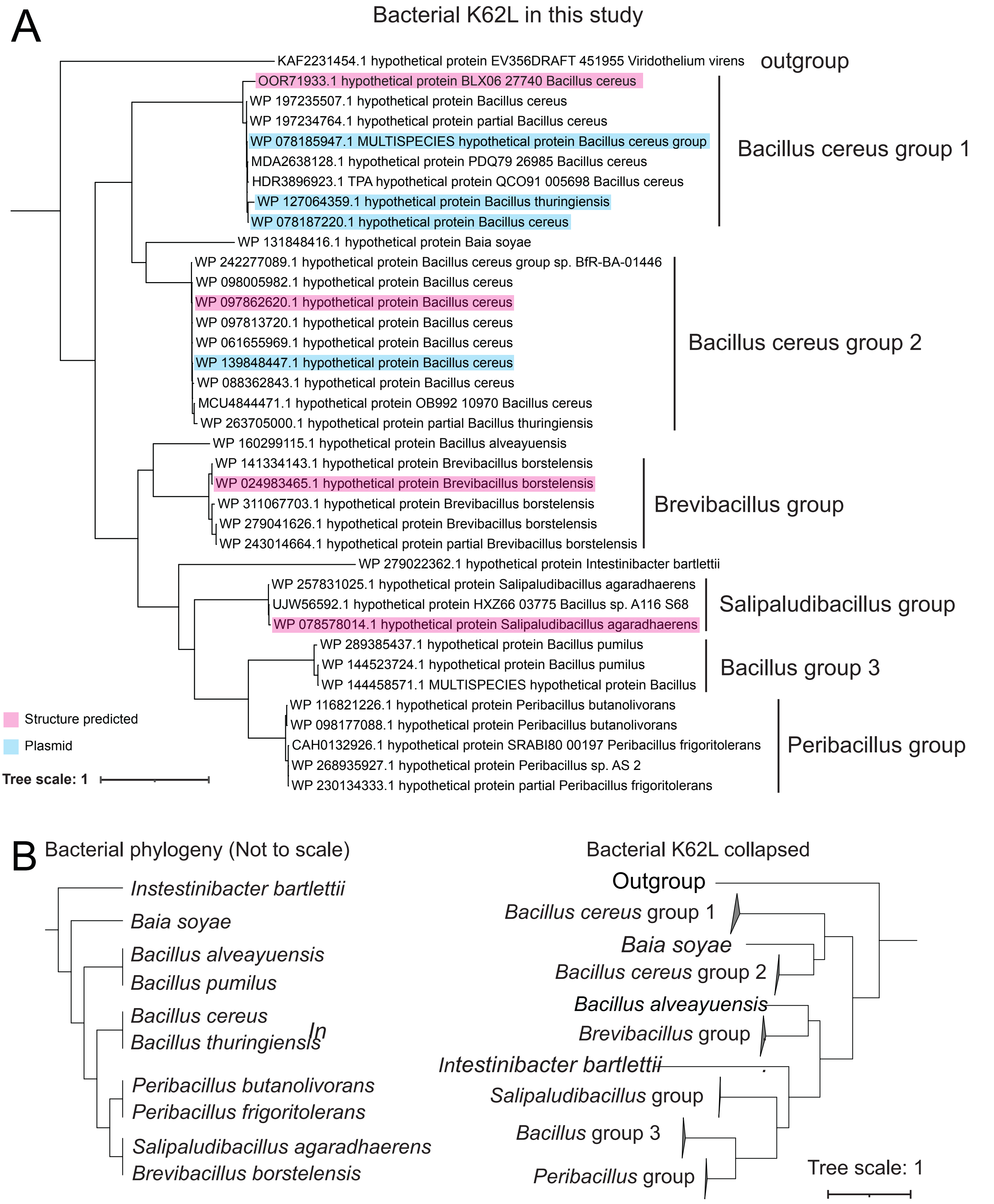

Figure S14

Phylogenetics of Bacterial K62L. (A) Bacterial clade extracted from the phylogeny displayed in Figure 7. AlphaFold2 structure predictions were made for the sequences highlighted in pink. K62L genes found to be encoded on plasmids are highlighted in blue. (B) Comparison of established bacterial phylogeny as a cladogram (left) with the collapsed phylogeny of K62L from panel A to highlight the discordance (right).

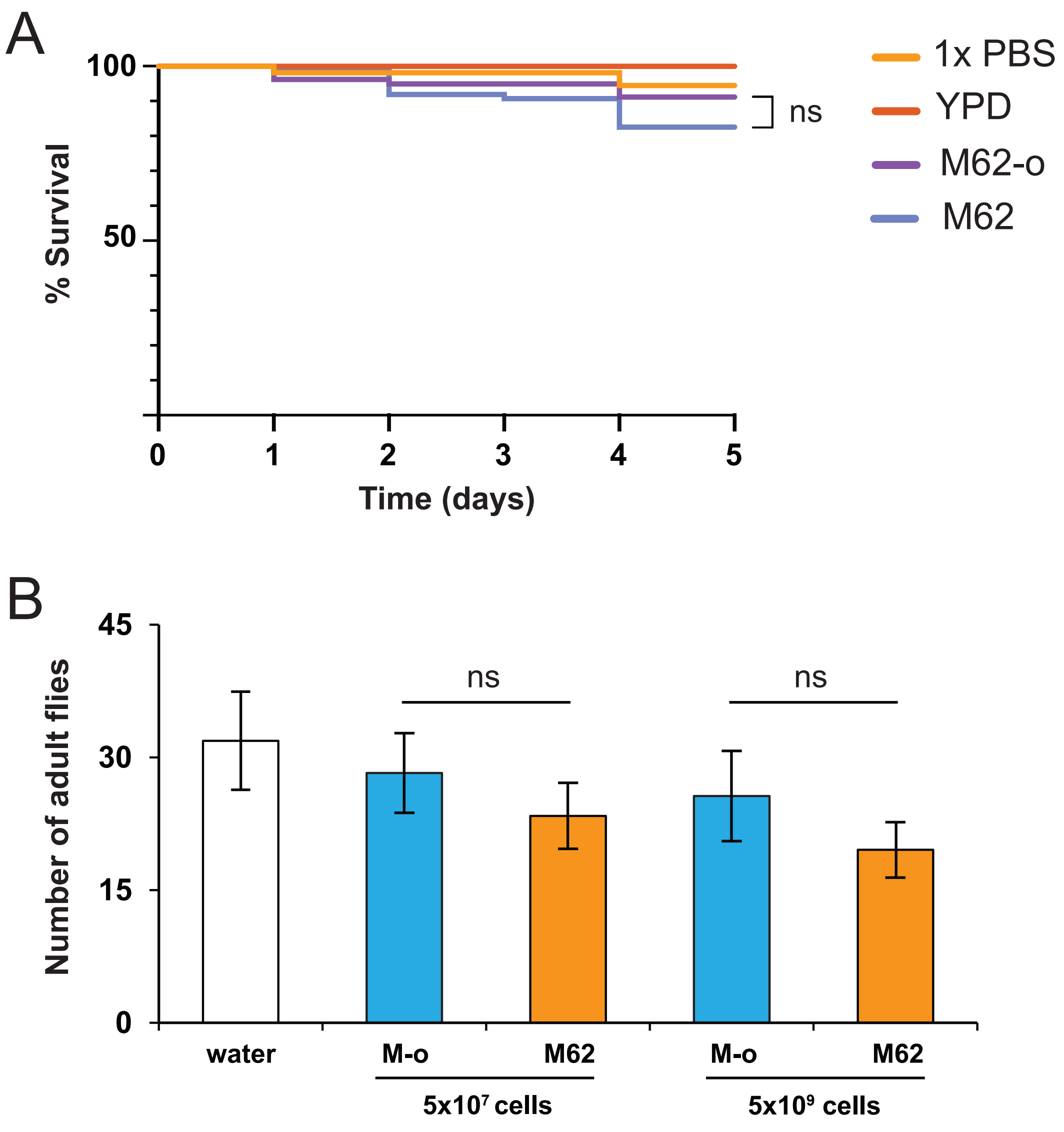

Figure S15

The K62 expression does not affect insect health or the virulence of yeasts. (A) The survival of *Galleria mellonella* larvae over five days after injection of either PBS, YPD, a K62 killer yeast (*S. paradoxus* OS169), or a cycloheximide-cured strain of OS169 lacking M62 (M62-o) log-rank (Mantel-Cox) test (ns = not significant). (B) The number of adult *Drosophila melanogaster* emerging after larvae were reared in food containing either water or the yeast *S. paradoxus* OS169 with (M62) or without (M62-o) killer toxin expression. Wilcoxon rank-sum test was used to identify statistically significant differences ($p < 0.05$) in adult fly counts between M62 and M62-o (ns = not significant).
