# Supplementary material for: The Saccharomyces killer toxin K62 is a protein of the aerolysin family
Source: mBio. 2025 Nov 11;16(12):e01425-25. doi: 10.1128/mbio.01425-25 (PMC12691639; doi:10.1128/mbio.01425-25)
Supplement: Legend — File S1 legend. [file mbio.01425-25-s0003.docx]

File S1. The phylogenetic relationship of the K62 family of proteins in Newick format.
