# Supplementary material for: The Saccharomyces killer toxin K62 is a protein of the aerolysin family
Source: mBio. 2025 Nov 11;16(12):e01425-25. doi: 10.1128/mbio.01425-25 (PMC12691639; doi:10.1128/mbio.01425-25)
Supplement: Supplementary tables — Tables S1-S14. [file mbio.01425-25-s0004.pdf]

Structure	Beta Sheets	Alpha Helices
SMKT (1KVD)	19%	16.7%
KP4 (1KPT)	27.6%	31.4%
WKT (1WKT)	48.6%	0%
KP6 (1KP6)	25.3%	35.4%
**Average**	**30.1% ± 12.8%**	**20.9% ± 16.1%**
K62 AF2 no signal seq.	41.6%	4.1%

Table S1. Percentage beta sheet and alpha helices in killer toxin crystal structures and K62 AlphaFold2 model. Assignment of secondary structure carried out in PyMol is defined by phi and psi angles of residues and hydrogen bonding of backbone atoms.

			Ramachandran					
	MolProb	Clash	Favored	Outliers	Rotamer outliers	Bad bonds	Bad angles	Twisted (non-Proline)
Unrelaxed	2.68	27.75	87.03%	7.53%	1.46%	64/1971	61/2706	1/230
100ns MD	1.62	0.84	94.95%	0.46%	4.59%	20/1959	119/2672	2/231

Table S2. SWISS-MODEL structure assessment scores of raw, unrelaxed AlphaFold2 output (unrelaxed) compared to the final frame of a 100 ns MD simulation.

Chain	Z	rmsd	lali	nres	%id	Description	Aero?	Species
2ztb-B	5.1	5.2	126	248	8	Crystal protein	**Yes**	*Bacillus thuringiensis*
8u5f-E	5	3.3	108	287	7	Heat-labile enterotoxin b chain	**Yes**	*Clostridium perfringens*
4lo4-B	4.6	5.7	113	421	4	Ha-70	No	-
6lh8-A	4.4	6.7	105	152	8	Aerolysin-like protein	**Yes**	*Bombina maxima*
1w3a-A	4.2	9.1	113	312	9	Hemolytic lectin Isla	**Yes**	*Laetiporus sulphureus*
2zs6-A	4	5.2	105	177	9	Hemagglutinin components ha3	No	-
4zno-A	3.8	8.2	121	318	7	Natterin-like protein	**Yes**	*Danio rerio*
3kog-A	3.8	9	123	226	7	Putative pore-forming toxin	**Yes**	*Phocaeicola vulgatus*
4jp0-A	3.5	4.3	110	378	4	Insecticidal crystal protein	**Yes**	*Bacillus thuringiensis*
6eu4-A	3.5	4.5	74	587	11	Tail spike protein	No	-
2d42-A	3.2	6.4	117	249	7	Non-toxic crystal protein	**Yes**	*Bacillus thuringiensis*
8bad-B	3.2	6.4	117	351	9	Binary toxin a-like protein	**Yes**	*Bacillus thuringiensis*
7ml9-A	3.1	8.3	134	295	8	Insecticidal protein	**Yes**	*Brevibacillus laterosporus*
4mjt-A	2.8	3.7	107	236	10	Monalysin	Yes	*Pseudomonas entomophila*
4m0h-A	2.7	12.7	81	216	6	Hypothetical protein, putative anti-sig	No	-
5efv-B	2.6	11.5	95	635	7	Phi eta orf 56-like protein	No	-
5mpd-U	2.4	11.3	91	298	7	26s proteasome regulatory subunit	No	-
3j8b-F	2.4	7.3	83	232	7	Translation initiation factor 3 subunit	No	-
4pkm-A	2.4	5.7	98	308	9	Cry51aa1	Yes	*Bacillus thuringiensis*
3g3l-A	2.2	4.9	104	291	6	Uncharacterized	No	-
1udx-A	2.2	2.2	41	412	15	The gtp-binding protein obg	No	-
3hsh-A	2.1	3.7	52	56	10	Collagen alpha-1(xviii) chain	No	-
5nxh-A	2.1	10.4	78	546	6	Long-tail fiber proximal subunit	No	-
8j0n-H	2	4.3	68	195	12	Er membrane complex subunit 1	No	-
5a5t-H	2	12.2	95	324	7	Translation initiation factor 3 subunit	No	-
2wjq-A	2	6.8	82	205	5	N-acetylneuraminic acid outer membrane	No	-

Table S3. DALI PDB25 results for K62 AlphaFold2 monomer final frame of 100 ns molecular dynamics simulation. Columns are labelled by the protein chain name, Z-score, RMSD, aligned length (lali), number of residues (nres), percentage identity (%id), a brief description of the proteins known function, whether the protein is of the aerolysin family of toxins (Aero?), and the species that the protein was isolated from (only including those that are aerolysins).

	Width	Inner diameter	Outer diameter
K62_6mer	133.1	22.4	39.6
K62_7mer	141.1	32.7	46.2
K62_8mer	189.6	44.7	56.7
K62_9mer	198.1	47.2	60.4
Parasporin2_6mer	160.3	23.0	45.6
Parasporin2_7mer	157.6	26.0	47.5
Parasporin2_8mer	168.3	32.0	53.9
Parasporin2_9mer	175.0	36.6	58.3
Aerolysin	154.3	26.3	47.0

Table S4. Dimensions of pre-pore oligomer structures of aerolysins and K62 (lacking signal sequences) in angstroms.

Pore	PDB	diameter	length
**Aerolysin pore**	5jzt	18.4	76.7
**Aerolysin quasi pore**	5jzw	27.0	70.9
Epsilon pore	6RB9	17.0	99.4
K62 7mer	n/a	29.5	34.3
K62 9mer	n/a	44.0	40.5

Table S5. Pore measurements in Angstroms.

Cysteine in **K62**	Aerolysin (CA)	Pore	**Pre-pore**	**monomer**
**C146-C271**	**V205-A423**	**9.4**	**9.9**	**n/a**
C201-C262	**P283-E415**	**8.4**	**9.8**	**10.0**
C227-C237	**V312-R397**	6.1	5.7	5.1

Table S6. Relative disulfide positions in aerolysin measured from alpha carbons in angstroms.

	**K62 oligomer**		
**Mutation**	**Monomer**	**Heptamer**	**Cleaved nonamer**
C146A	5.7	4.5	4
C201A	3.0	6.0	2.9
C227A	0.7	2.8	2.7
C237A	0.2	3.7	3.6
C262A	3.0	5.0	5.5
C269A	-0.3	-0.3	0.0
C271A	6.1	6.9	4.3

Table S7. FOLDX 5.0 ΔΔG values of K62 cysteine mutants. Using a cutoff of 2 kcal/mol, blue indicates a destabilizing mutation, white indicates no effect, and yellow indicates a stabilizing mutation.

**Species**	**Accession**	**pLDDT**	**RMSD to K62**
M62 satellite	ATN38496	77.0	0.0
Cutaneotrichosporon cavernicola	BEI87225	80.1	2.7
Maudiozyma barnettii	CAD1782901	70.8	2.7
Peribacillus frigoritolerans	CAH0132926	79.9	5.0
Saccharomyces cerevisiae	CAI4293746	65.5	2.8
Millerozyma farinosa	CCE78608	71.4	3.7
Millerozyma farinosa	CCE88410	77.0	3.1
Pyrenophora teres	EFQ96194	77.4	7.9
Fusarium oxysporum	EWY87637	78.9	3.2
Fusarium oxysporum	EWY95052	66.7	9.8
Fusarium oxysporum	EXK79730	74.2	3.4
Ambrosiozyma monospora	GME70772	66.3	3.7
Coniochaeta sp. 2T2.1	KAB5515543	68.5	11.3
Colletotrichum asianum	KAF0331288	80.7	2.9
Sporormia fimetaria	KAF2742077	75.8	5.2
Fusarium acutatum	KAF4415336	72.5	7.1
Fusarium acutatum	KAF4430519	76.9	8.5
Fusarium bulbicola	KAF5974755	84.1	6.8
Metschnikowia pulcherrima	KAF7999827	88.4	3.5
Acephala macrosclerotiorum	KAF8861807	77.0	10.8
Fusarium xylarioides	KAG5758416	85.0	3.4
Parastagonospora nodorum	KAH5607816	58.9	4.9
Fusarium avenaceum	KAH6970216	85.4	3.1
Nakaseomyces glabratus	KAH7591390	70.5	3.1
Xylariales sp. PMI_506	KAH8662392	73.5	5.9
Nemania serpens	KAI1195413	72.2	3.1
Adiantum capillus-veneris	KAI5081312	68.1	2.8
Septoria linicola	KAI5357779	74.2	2.9
Candida parapsilosis	KAI5901210	66.5	3.8
Nakaseomyces glabratus	KAI8390687	78.6	1.9
Cladochytrium replicatum	KAI8808066	81.6	3.7
Lecanicillium fungicola	KAJ2975367	80.8	3.5
Lecanicillium fungicola	KAJ2976130	81.9	4.2
Lecanicillium fungicola	KAJ2977295	58.5	4.0
Diphasiastrum complanatum	KAJ7517662	66.0	3.8
Candidozyma auris	KND99538	77.9	9.7
Adiantum nelumboides	MCO5568161	62.1	3.5
Adiantum nelumboides	MCO5602774	59.5	3.8
Eremothecium gossypii	NP_986301	76.5	2.3

Bacillus cereus	OOR71933	73.1	4.2
Aspergillus taichungensis	PLN78462	76.2	3.6
Elsinoe australis	PSK33493	82.0	4.0
Elsinoe australis	PSK53113	72.7	2.4
Metschnikowia aff. pulcherrima	QBM89989	81.4	3.6
Hortaea werneckii	RMX81907	69.4	4.5
Maudiozyma saulgeensis	SMN20996	75.9	3.2
Brevibacillus borstelensis	WP_024983465	80.9	6.5
Salipaludibacillus agaradhaerens	WP_078578014	82.2	7.4
Bacillus cereus	WP_097862620	80.9	2.6
Scheffersomyces stipitis	XP_001383827	76.2	4.1
Scheffersomyces stipitis	XP_001384150	70.6	5.7
Verticillium dahliae	XP_009656760	74.7	4.6
Trichosporon asahii	XP_014177264	86.2	4.6
Aspergillus nomiae	XP_015404001	76.7	3.0
Debaryomyces fabryi	XP_015464483	69.3	4.8
Candida parapsilosis	XP_036663347	73.5	3.1
Candida margitis	XP_051674245	71.6	3.2
Colletotrichum costaricense	XP_060314141	69.5	3.4
Colletotrichum abscissum	XP_060402588	83.7	2.7

Table S8. RMSD table of representative K62L AlphaFold2 model predictions

NCBI Accession	SignalP6.0	RMSD*	global pLDDT	Length (aa)
OOR71933.1	no confidence	2.82	73.11	213
WP_097862620.1	25-26 aa (1.00)	4.51	80.94	252
WP_078578014.1	no confidence	4.26	82.18	237
WP_024983465.1	27-28 aa (1.00)	4.06	80.93	237
MDN6570107.1	no confidence	6.17	65.49	320

Table S9. Statistics of representative structures of bacterial K62Ls. SignalP6.0 was used to predict the signal sequence of each protein. The location of the proteolytic cleavage is noted by its amino acid position, and the prediction confidence is in parentheses. Using the full-length bacterial model, RMSD was determined by cealign to the K62 model. pLDDT illustrates that each model is of moderate confidence. Length is the protein-coding sequence. *RMSD to noSS_K62 by cealign of full structures

EXL39644.1	(Foxy)	100.00								
RGP81422.1	(Flon)	26.15	100.00							
KAH7232946.1	(Ftri)	28.77	90.75	100.00						
KAK2477225.1	(Foxy)	27.98	92.07	96.48	100.00					
XP_031052436.1	(Fodo)	28.44	91.19	96.04	97.8	100.00				
KAK2674048.1	(Foxy)	28.44	91.63	95.59	98.24	98.24	100.00			
SPJ76703.1	(Ftor)	28.44	91.63	95.59	97.36	97.36	97.8	100.00		
BDU14757.1	(Fcom)	27.52	92.07	96.04	97.80	97.36	97.80	97.80	100.00	
KAG7414062.1	(Foxy)	27.98	92.51	96.48	98.24	97.8	98.24	98.24	99.56	100.00

Table S10. Pairwise percentage amino acid identity table of selected *Fusarium* K62L. The first column represents the NCBI accession number of each *Fusarium* species compared: *F. oxysporum* (Foxy), *F. longipes* (Flon), *F. tricinctum* (Ftri), *F. odoratissimum* (Fodo), *F. torulosum* (Ftor), and *F. commune* (Fcom).

**Accession**	**Strain**	**Plasmid, size (bp)**	**Accession of most closely related, Amino acid ID, Nucleotide ID**
WP_127064359.1	*Bacillus thuringiensis* C15	pBMB172, 172,221	WP_078187220.1, 94.85%, 97.44%
WP_078187220.1	*Bacillus cereus* CPT56D-587-MTF	unnamed5, 72,739	WP_127064359.1, 94.85%, 97.44%
WP_078185947.1	*Bacillus thuringiensis* C15	pBMB240, 240,314	MDA2638128.1, 99.14%, 99.72%
WP_139848447.1	*Bacillus cereus* A22	unnamed1, 480,744	WP_088362843.1, 98.81%, 98.95%
WP_139848447.1	*Bacillus cereus* FORC087	pFORC087.1, 512,032	WP_088362843.1, 98.81%, 98.95%

Table S11. Bacterial K62L on plasmids

Species	Strain	Genotype
S. cerevisiae	BY4741	*his3Δ1, leu2Δ0, met15Δ0, ura3Δ0*
S. cerevisiae	CRY	MATa *ade2-1 ura3-1 leu2-3,112 trp1 his3-11*
S. paradoxus	OS169	n/a
E. coli	10-Beta	*Δ(ara-leu) 7697 araD139 fhuA ΔlacX74 galK16 galE15 e14- ϕ80dlacZΔM15 recA1 relA1 endA1 nupG rpsL (StrR) rph spoT1 Δ(mrr-hsdRMS-mcrBC)*
E. coli	BL21	*F- ompT* $hsdS_B(r_B^- m_B^-)$ *gal dcm* (DE3) pLysS (Cm^R)
S. cerevisiae	MSY114	n/a
N. glabratus	EF0616blo1	n/a
N. glabratus	CST 109	n/a
S. cerevisiae	Y-1891	n/a
N. glabratus	I 1718	n/a
S. cerevisiae	NCYC 738	n/a
S. bayanus	CBS7001	n/a
S. kudriavzevii	NBRC1802	n/a
S. mikatae	NBRC1815	n/a
S. cerevisiae	Y-2429	n/a
S. cerevisiae	DSM 70459	n/a
S. cerevisiae	CYC 1113	n/a
N. dairenensis	NCYC_777	n/a
S. paradoxus	OS294	n/a
S. cerevisiae	NCYC1001	n/a
S. cerevisiae	San Diego Super	n/a
S. cerevisiae	AEA	n/a
S. cerevisiae	Voss KveiK	n/a
S. cerevisiae	WLP001	n/a
S. paradoxus	CBS432	n/a
Y. lipolytica	O38	n/a
N. castellii	NCYC2898	n/a
S. cerevisiae	CYC 1058	n/a
S. cerevisiae	CYC 1102	n/a
S. cerevisiae	NCYC 1006	n/a
S. cerevisiae	CYC 1170	n/a
S. cerevisiae	CYC 1172	n/a
S. cerevisiae	DBVPG 1373	n/a
S. cerevisiae	DBVPG 6765	n/a
S. cerevisiae	FY 4	n/a
S. cerevisiae	K12	n/a
S. cerevisiae	MS 300c	n/a
S. cerevisiae	BJH 001	n/a
S. cerevisiae	1116	n/a
S. cerevisiae	Y-5509	n/a
S. cerevisiae	Y-27106	n/a
S. cerevisiae	Y-27788	n/a
S. cerevisiae	YB-432	n/a
K. africanus	NCYC 2729	n/a

Table S12. Microorganisms used in the current study.

Primer	Sequence	Purpose
JC006F	gccggattatgcgTAAACCCAGCTTTCTTGTACAAAG	K62 HA C terminal tag
JC006R	acatcatacggataGCTGCACCGACAGTTAGC	K62 HA C terminal tag
JC015F	GTATCAACAGgcaGTGATGTGGGC	K62 SDM R216A
JC015R	CAGGCCTGACCTACACTC	K62 SDM R216A
C146A F	AATATCACCCgctGCAAAGTTTGGATTAAGTGACC	K62 SDM C146A
C146A R	GGATACCAGGGTGACCAC	K62 SDM C146A
C201A F	GACCTTGACGgctAACGTCGGTGC	K62 SDM C201A
C201A R	CCGCTATACGACAAGGATTG	K62 SDM C201A
C227A F	GAAACAATACgctCGACTCAACACCAGTG	K62 SDM C227A
C227A R	TGCATATCAGCCCACATC	K62 SDM C227A
C237A F	GAAGACTGTGgctGACGCTTGGAGCC	K62 SDM C237A
C237A R	CCACTGGTGTTGAGTCG	K62 SDM C237A
C262A F	TATTGTAGGTgctAGTACCGGTGATGCTAAC	K62 SDM C262A
C262A R	CGATCAGTGGCAGTTTGAC	K62 SDM C262A
JC034F	TGATGCTAACgcgCGGTGCAGCTATCC	K62 SDM C269A
JC034R	CCGGTACTGCAACCTAC	K62 SDM C269A
JC035F	TAACTGTCGGgcgAGCTATCCGTATG	K62 SDM C271A
JC035R	GCATCACCGGTACTG	K62 SDM C271A

Table S13. Primers used in the current study

**Name**	**Description**	**Marker**
pENTR SpK62	Entry vector containing K62	Kan
pAG426-GAL-ccdB	High copy vector for inducible yeast expression	Amp/Cm/*URA3*
pAG306-GAL-ccdB	Integrative vector for inducible yeast expression	Amp/Cm/*URA3*
pDEST14	Destination vector for *E. coli* expression	Amp/Cm
pENTR K62-HA	Entry vector plasmid with C-terminal HA tag	Kan
pENTR K62-HA R216A	pENTR SpK62 (R216A) with C-terminal HA tag	Kan
pENTR K62 C146A	pENTR SpK62 (C146A)	Kan
pENTR K62 C201A	pENTR SpK62 (C201A)	Kan
pENTR K62 C227A	pENTR SpK62 (C227A)	Kan
pENTR K62 C237A	pENTR SpK62 (C237A)	Kan
pENTR K62 C262A	pENTR SpK62 (C262A)	Kan
pENTR K62-HA C269A	pENTR SpK62 (C269A) with C-terminal HA tag	Kan
pENTR K62-HA C271A	pENTR SpK62 (C271A) with C-terminal HA tag	Kan
pENTR K62 C146A C271A	pENTR SpK62 (C146A/C271A)	Kan
pENTR K62 C201A C262A	pENTR SpK62 (C201A/C262A)	Kan
pENTR K62 C227A C237A	pENTR SpK62 (C227A/C237A)	Kan
pAG306GAL K62	pAG306-GAL-ccdB K62	Amp/*URA3*
pAG306GAL K62-HA	pAG306-GAL-ccdB K62-HA	Amp/*URA3*
pAG426GAL K62-HA	pAG426-GAL-ccdB K62-HA	Amp/*URA3*
pAG426-GAL K62-HA R216A	pAG426-GAL-ccdB /w K62-HA (R216A)	Amp/*URA3*
pAG306GAL K62 C146A	pAG306-GAL-ccdB K62-HA SpK62 (C146A)	Amp/*URA3*
pAG306GAL K62 C201A	pAG306-GAL-ccdB K62-HA SpK62 (C201A)	Amp/*URA3*
pAG306GAL K62 C227A	pAG306-GAL-ccdB K62-HA SpK62 (C227A)	Amp/*URA3*
pAG306GAL K62 C237A	pAG306-GAL-ccdB K62-HA SpK62 (C237A)	Amp/*URA3*
pAG306GAL K62 C262A	pAG306-GAL-ccdB K62-HA SpK62 (C262A)	Amp/*URA3*
pAG306GAL K62-HA C269A	pAG306-GAL-ccdB K62-HA SpK62 (C269A)	Amp/*URA3*
pAG306GAL K62-HA C271A	pAG306-GAL-ccdB K62-HA SpK62-HA (C271A)	Amp/*URA3*
pAG306GAL K62 C146A C271A	pAG306-GAL-ccdB K62-HA SpK62 (C146A/C271A)	Amp/*URA3*
pAG306GAL K62 C201A C262A	pAG306-GAL-ccdB K62-HA SpK62 (C201A/C262A)	Amp/*URA3*
pAG306GAL K62 C227A C237A	pAG306-GAL-ccdB K62-HA SpK62 (C227A/C237A)	Amp/*URA3*
pDEST14 K62-HA	pDEST14 K62-HA (94-843 bp)	Amp

Table S14. Plasmids used in the current study.
